# Environmental factors influence the local establishment of *Wolbachia* in *Aedes aegypti* mosquitoes in two small communities in central Vietnam

**DOI:** 10.12688/gatesopenres.13347.2

**Published:** 2022-04-07

**Authors:** Nguyen T. Hien, Dang D. Anh, Nguyen H. Le, Nguyen T. Yen, Tran V. Phong, Vu S. Nam, Tran N. Duong, Nguyen B. Nguyen, Duong T.T. Huong, Luu Q. Hung, Chau N.T. Trinh, Nguyen V. Hoang, Vien Q. Mai, Le T. Nghia, Nguyen T. Dong, Le H. Tho, Simon Kutcher, Tim P. Hurst, Jacqui L. Montgomery, Megan Woolfit, Edwige Rances, Peter Kyrylos, Katherine L. Anders, Le Nguyen, Jack Brown-Kenyon, Angela Caird, Breeanna J. McLean, Inaki Iturbe-Ormaetxe, Scott A. Ritchie, Scott L. O'Neill, Peter A. Ryan

**Affiliations:** 1 National Institute of Hygiene and Epidemiology, Hanoi, Vietnam; 2 Institute Pasteur, Nha Trang, Vietnam; 3 Khanh Hoa Health Department, Nha Trang, Vietnam; 4 World Mosquito Program, Institute of Vector-Borne Disease, Monash University, Clayton, Victoria, 3800, Australia

**Keywords:** Dengue, World Mosquito Program, Wolbachia, Aedes aegypti, mosquito release

## Abstract

**Background: **The *w*Mel strain of *Wolbachia* has been successfully introduced into *Aedes aegypti* mosquitoes and subsequently shown to reduce transmission of dengue and other pathogens, under both laboratory and field conditions. Here we describe the entomological outcomes of *w*Mel *Wolbachia* mosquito releases in two small communities in Nha Trang City in central Vietnam.

**Methods: **The *w*Mel strain of *Wolbachia *was backcrossed into local *Aedes aegypti* genotype and mosquito releases were undertaken by community members or by staff. Field monitoring was undertaken to track *Wolbachia* establishment in local *Ae. aegypti* mosquito populations. Ecological studies were undertaken to assess relationships between environmental factors and the spatial and temporal variability in *Wolbachia* infection prevalence in mosquitoes.

**Results: **Releases of *w*Mel *Wolbachia Ae. aegypti* mosquitoes in two small communities in Nha Trang City resulted in the initial establishment of *Wolbachia* in the local *Ae. aegypti* mosquito populations, followed by seasonal fluctuations in *Wolbachia* prevalence. There was significant small-scale spatial heterogeneity in *Wolbachia* infection prevalence in the Tri Nguyen Village site, resulting in the loss of *w*Mel *Wolbachia *infection in mosquitoes in north and center areas, despite *Wolbachia* prevalence remaining high in mosquitoes in the south area. In the second site, Vinh Luong Ward, *Wolbachia* has persisted at a high level in mosquitoes throughout this site despite similar seasonal fluctuations in *w*Mel *Wolbachia *prevalence.

**Conclusion: **Seasonal variation in *Wolbachia* infection prevalence in mosquitoes was associated with elevated temperature conditions, and was possibly due to imperfect maternal transmission of *Wolbachia*. Heterogeneity in *Wolbachia* infection prevalence was found throughout one site, and indicates additional factors may influence *Wolbachia* establishment.

## Introduction

*Aedes aegypti* mosquitoes containing the *w*Mel *Wolbachia* strain have been shown to have a reduced ability to transmit a range of viruses including dengue, Zika, chikungunya, yellow fever and Mayaro viruses ( Ryan *et al.*, 2020). Field trials involving releases of *w*Mel *Wolbachia* infected *Ae. aegypti* mosquitoes have shown that *Wolbachia* can be deployed and established in local mosquito populations ( Garcia *et al.*, 2019; Gesto *et al.*, 2021; Hoffmann *et al.*, 2011; Hoffmann *et al.*, 2014; Indriani *et al.*, 2020; O'Neill *et al.*, 2019; Ryan *et al.*, 2020; Schmidt *et al.*., 2017; Tantowijoyo *et al.*, 2020; Utarini *et al.*, 2021). The *w*Mel *Wolbachia* infection has been shown to persist in local mosquito populations ( Gesto *et al.*, 2021; O'Neill *et al.*, 2019; Ryan *et al.*, 2020; Tantowijoyo *et al.*, 2020; Utarini *et al.*, 2021), and the viral blocking properties remain stable ( Carrington *et al.*, 2018; Frentiu *et al.*, 2014; Gesto *et al.*, 2021). In areas where *w*Mel *Wolbachia* has been established in local mosquito populations, dengue incidence has been significantly reduced, resulting in near elimination of local dengue transmission in northern Australia ( O'Neill *et al.*, 2019; Ryan *et al.*, 2020); 73% reduction in dengue incidence in a quasi-experimental trial in Yogyakarta, Indonesia ( Indriani *et al.*, 2020); 77.1% reduction in dengue incidence in a cluster randomized trial in Yogyakarta, Indonesia ( Utarini *et al.*, 2021); and 69% reduction in dengue incidence, 56% reduction in chikungunya incidence, and 37% reduction in Zika incidence, in Niterói, Brazil ( Pinto *et al.*, 2021).

Here we describe the entomological outcomes of *w*Mel *Wolbachia* mosquito releases in two small communities in Nha Trang City in central Vietnam. These releases resulted in the initial establishment of *w*Mel *Wolbachia* in the local *Ae. aegypti* mosquito populations, followed by seasonal fluctuations in *Wolbachia* infection prevalence in *Ae. aegypti* mosquitoes. In the Tri Nguyen Village site we observed significant small-scale spatial heterogeneity in *Wolbachia* infection prevalence, with localized losses of infection in north and central areas but high prevalence in the south area. Despite similar overall climatic conditions and seasonal fluctuations in *w*Mel *Wolbachia* prevalence in the second site, Vinh Luong Ward, *Wolbachia* has persisted at a high level in mosquitoes throughout this site. We investigate the environmental factors that may be associated with the observed fluctuations in *Wolbachia* infection prevalence in mosquitoes in these sites.

## Methods

### Intervention area

**Figure 1.**
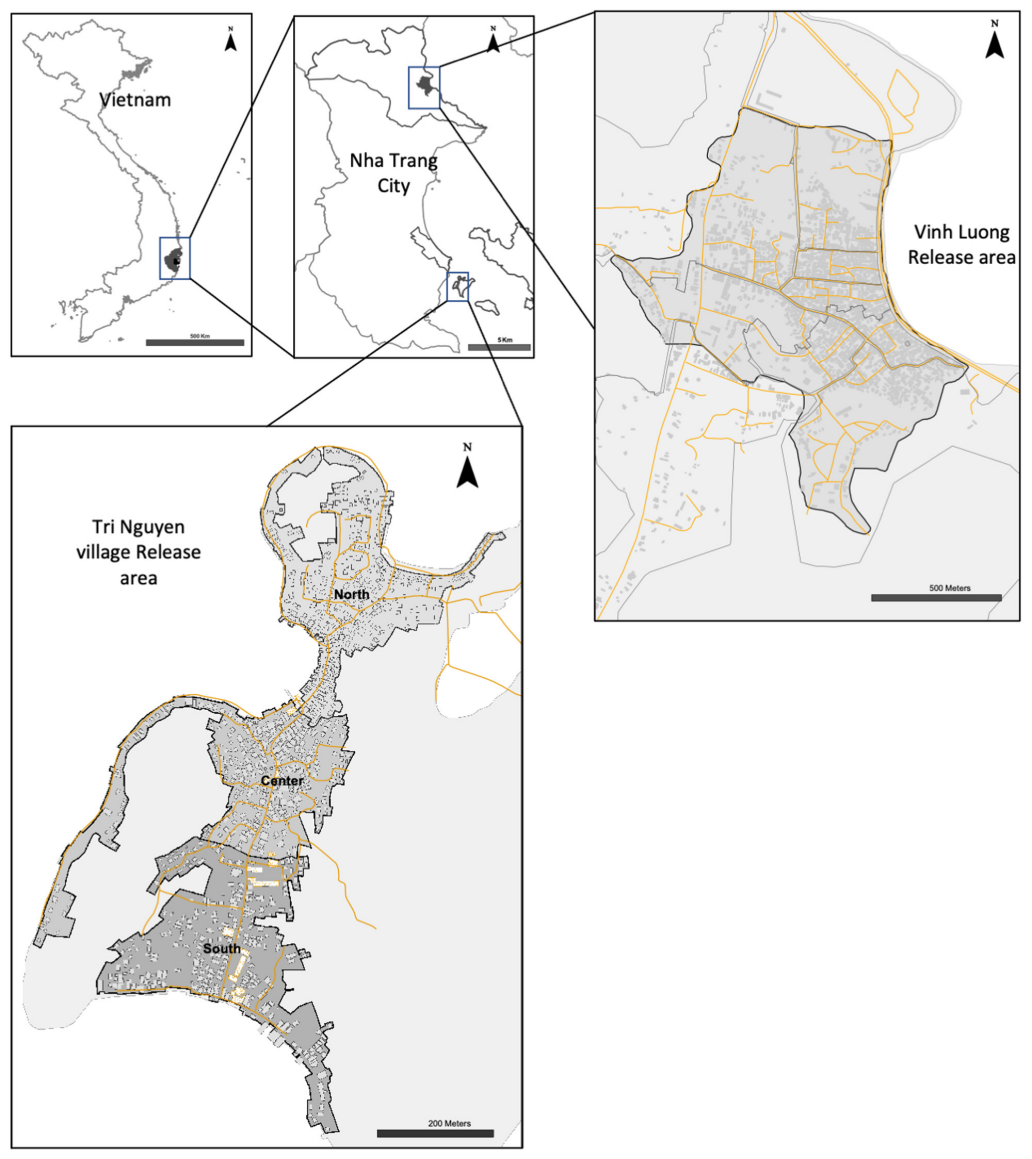
Map of Tri Nguyen village and Vinh Luong release areas in Nha Trang City, Vietnam.

**Figure 2.**
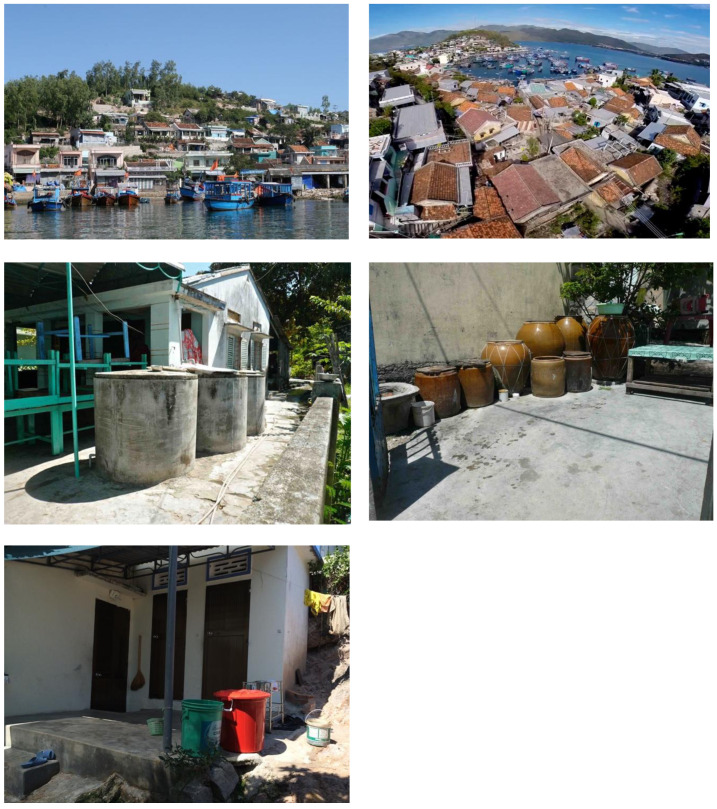
Tri Nguyen village.

*Tri Nguyen village.* Releases occurred in Tri Nguyen village, a fishing community located on Hon Mieu Island, Nha Trang City, in central Vietnam. The island is located 1 km from Nha Trang City, and is approximately 1.2 km ^2^ (117 ha) in size. The densely populated area on Tri Nguyen village is approximately 0.22 km ^2^ (22 ha) in size and is comprised of 821 households (population 3,527), located in a rough north-south pattern on the western side of the island ( Figure 1, Figure 2).

**Figure 3.**
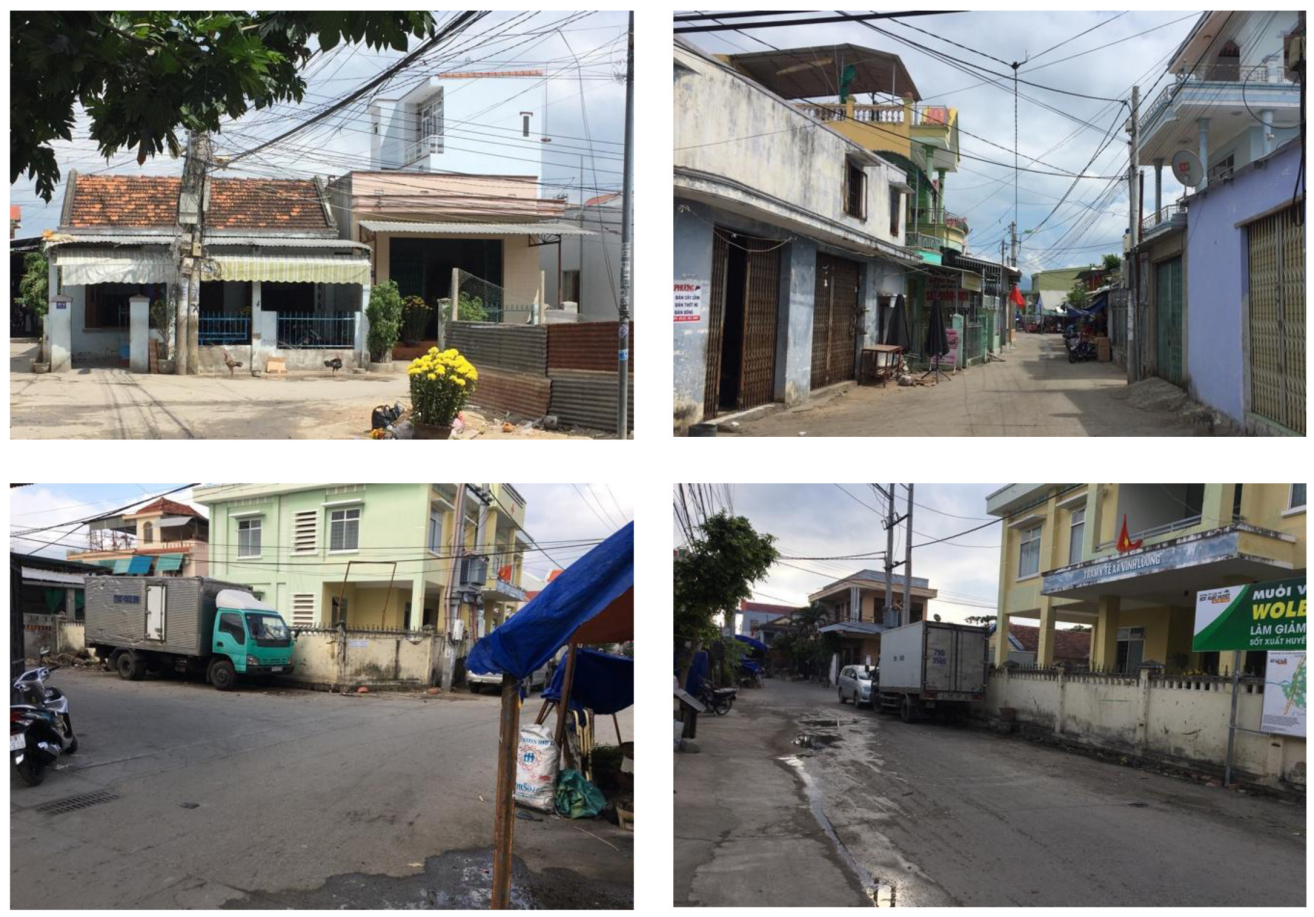
Vinh Luong Ward.

*Vinh Luong Ward.* Vinh Luong Ward is a fishing community located approximately 10 km north of the Nha Trang City center. The release area of 1.0 km ^2^ was comprised of eight hamlets in the central residential area with a population of 12,143 in 2,846 households ( Figure 1, Figure 3).

### Rearing

For the Tri Nguyen releases in 2014, a local *w*Mel *Ae. aegypti* line was created by mating infected virgin females from a Cairns, Australia *w*Mel-infected *Ae. aegypti* line (described in Walker *et al.*, 2011) to uninfected males from Tri Nguyen for six generations ( Table 1). The uninfected wild-type mosquitoes were collected as larvae or pupae from water holding containers, or as eggs from ovitraps ( Ritchie, 2001), from households in Tri Nguyen village. To minimize laboratory adaptation after backcrossing, male *Ae. aegypti* from field collected *Ae. aegypti* (F1 eggs) obtained from Tri Nguyen village as described above, were introduced into the colony each generation, so that they constituted 10% of the new male population. Two colonies (release stock and back-up) were maintained in two insectaries located at the National Institute of Hygiene and Epidemiology (NIHE), Hanoi, Vietnam. Both colonies had 30 cages (30 x 30 x 30 cm), stocked at a density of around 400 females, and were bloodfed on human volunteers weekly. Volunteer blood feeders were sourced from institutional (NIHE) staff or their colleagues and were excluded if their temperature was 38°C or above, if they had been taking antibiotics in the last five days or if they had been experiencing a febrile illness. Volunteers provided an exposed arm or leg to a cage of mosquitoes for 10 minutes (maximum of three cages per volunteer), with each cage of mosquitoes exposed only to a single volunteer. Eggs were collected from containers lined with filter paper, with each cage producing approximately 6,000 eggs. Egg strips were removed from cages and placed onto adsorbent paper towel and stored in sealed plastic bags for three days at 27°C, after which time they were removed from the plastic bags and paper towel and were then dried under insectary conditions (27°C, 80% relative humidity) for 90–120 minutes. Dried egg strips were then packed between sheets of filter paper and transferred to sealed plastic bags each containing a 2 x 3 cm piece of moistened filter paper to maintain moisture and prevent desiccation of the eggs. The sealed plastic bags containing the egg strips were placed into insulated containers and were shipped under ambient temperature conditions to Institute Pasteur Nha Trang (IPNT) via courier.

**Table 1.** *w*Mel *Wolbachia Aedes aegypti* release lines for Tri Nguyen and Vinh Luong sites.

Release line	Characteristic	Description			
Tri Nguyen	Backcrossing source	Tri Nguyen			
	Backcrossing method	Six generations of backcrossing, followed by introduction of 10% wild type males (F1) per generation			
	*Wolbachia* infection rate	qPCR screening of subsample of larvae from each generation of colony material (minimum *Wolbachia* prevalence of > 97%)			
	Egg hatch rate (Pre-release)	Sample of colony eggs from n = 6 cages, hatch rate assessed for each cage Mean hatch rate 96.4% +/- 3.7% (s.d.)			
	Fecundity (Release)	Eggs from n = 50 females, fecundity and hatch rate assessed for each female 52.2 +/- 12.4% (s.d.)			
	Hatch rate (Release)	72.2% +/- 12.5% (s.d.)			
	Maternal transmission (Release)	n = 50 females Mean infection rate in progeny 100.0% +/- 0.0% (sd)			
Vinh Luong	Backcrossing source	Nha Trang City urban area (F1)			
	Backcrossing Method	Six generations of backcrossing, followed by introduction of 10–20% wild type males (F1) per generation			
	*Wolbachia* infection rate (Pre- release and release)	qPCR screening of 172 adult mosquitoes from each generation of colony material Average *Wolbachia* infection rate 100% +/- 0.0% (s.d.)			
	Fecundity (Pre-release)	n = 50 females Mean 71.5 +/- 16.5 (sd) eggs per female			
	Egg hatch rate (Pre-Release)	Sample of eggs from n = 50 females, hatch rate assessed for each female Mean 78.5% +/- 22.8% (sd)			
	Maternal transmission (Pre-release)	n = 50 females Mean infection rate in progeny 100.0% +/- 0.0% (sd)			
	Insecticide resistance	Vinh Luong Release line (Pre-release)		Nha Trang City urban (F1)	
	**Insecticide**	**Mean Mortality (%)**	**(sd)**	**Mean Mortality (%)**	**(sd)**
	Malathion (0.8%)	11.0	(5.5)	4.0	(4.2)
	Malathion (5.0%)	100.0	(0)	99.0	(2.2)
	Bendiocarb (0.1%)	74.0	(6.5)	66.0	(6.5)
	Bendiocard (0.5%)	100.0	(0)	100.0	(0)
	Permethrin (0.25%)	1.0	(2.2)	8.0	(7.6)
	Permethrin (1.25%)	3.0	(2.7)	17.0	(11.0)
	Deltamethrin (0.03%)	9.0	(2.2)	18.0	(7.6)
	Deltamethrin (0.15%)	67.0	(10.4)	76.0	(9.6)

For quality assurance of the mosquito colonies, a total of 10 adult mosquitoes were randomly sampled from cages at four to five days after blood-feeding, and were screened for DENV and CHIK by qRT-PCT ( Quyen *et al.*, 2018). Primer and probe sequences are as follows; pan-DENV F: AAGGACTAGAGGTTAGAGGAGACCC and R: CGTTCTGTGCCTGGAATGATG, with probe 5’-Lc640 (or Cy5)- AACAGCATATTGACGCTGGGAGAGACCAGA- Iowablack -3’ and CHIKV F: 5’-AAGCTYCGCGTCCTTTACCAAG3’, R: 5’-CCAAATTGTCCYGGTCTTCCT-3’ with probe 5’-HEX-CCAATGTCYTCNGCCTGGACACCTT- BHQ1 -3’. RNA underwent one freeze-thaw cycle with qRT-PCR reaction performed using the Lightcycler Multiplex RNA Virus Master kit (Roche) with the following conditions; 50 °C for 10 mins, 95 °C for 30 sec, followed by 45 cycles of 95 °C for 3 sec, 60 °C for 30 sec, 72 °C for 1 sec and 1 cycle of 40 °C for 1 sec.

For *Wolbachia* screening, a random sample of larvae and adult females were tested for *w*Mel infection by Taqman qPCR each week ( Dar *et al.*, 2008; O’Neill *et al.*, 2019; Yeap *et al.*, 2014), with a minimum acceptable *Wolbachia* prevalence of 97%. qPCR was undertaken using the Lightcycler 480 Probes Master (Roche) kit with cycling conditions; x1 95°C for 5 minutes, x45 95°C for 10 seconds, 60°C for 15 seconds, 72°C for 1 second with single acquisition and x1 40°C for 10 seconds. *Wolbachia* was detected using either TM513 (primers F: 5’-CAAATTGCTCTTGTCCTGTGG -3’, R: 5’-GGGTGTTAAGCAGAGTTACGG-3’ and probe 5’-LC640 -TGAAATGGAAAAATTGGCGAGGTGTAGG-iowaBlack-3’) or WSP primers (F: 5’-CATTGGTGTTGGTGTTGGTG-3’, R: 5’-ACACCAGCTTTTACTTGACCAG-3’ with probe: 5’-LC640-TCCTTTGGAACCCGCTGTGAATGA-IowaBlack-3’) and *Ae. aegypti* rps17 reference detected with primers F: 5'-TCCGTGGTATCTCCATCAAGCT-3', R: 5'-CACTTCCGGCACGTAGTTGTC-3' and probe 5'FAM- CAGGAGGAGGAACGTGAGCGCAG-BHQ1-3). All qPCR and qRT-PCR testing were undertaken using the LightCycler 480 Instrument II - Roche Life Science.

The *Wolbachia* mosquito line was characterized in terms of key fitness traits including adult female fecundity, egg hatch rate, and *Wolbachia* maternal transmission efficiency using previously described methods ( Walker *et al.*, 2011; Yeap *et al.*, 2011) ( Table 1). Fecundity was assessed using multiple human blood feeders with a total of 50 bloodfed female mosquitoes. Females were transferred into individual 40mL tubes containing water and filter paper, for oviposition. Females were allocated seven days in oviposition tubes until they were examined for the presence or absence of eggs, at which time the eggs were counted to determine fecundity. Hatch rates of eggs were determined by transferring paper and water from oviposition tubes into trays containing 250mL of water and a small amount of larval diet as described below. Eggs were left for 48 hours to hatch before the larvae in each tray were counted. As some eggs may not have matured during the first hatch, egg papers were dried down and stored for three days before immersing a second time. The numbers of larvae from the first and second hatch were combined to determine the hatch rate of eggs. For maternal transmission assessments, *Wolbachia* infected virgin females were mated with wild-type F0 or F1 males over a 24 h period. After 24 h a human blood meal was provided and individual females that appeared fully engorged were placed into individual oviposition cups. Each cup was lined with a moistened piece of filter paper as a medium for oviposition. After 24 hrs following oviposition, the female mosquitoes were collected and stored in 70% ethanol, and the eggs were counted and then conditioned for three days, prior to hatching in water containing a small amount of larval diet as described below. After 24 hours the number of hatched larvae were recorded. Larvae were reared until they reached II-IV instar then transferred to 70% ethanol. Adult females and progeny (n=10–20) were processed for *Wolbachia* infection using a Taqman qPCR assay as described above.

 Egg viability was also monitored to determine the effectiveness of egg storage and incubation methods as well as any effects of transport between the release stock colony at NIHE (Hanoi) and the rearing facility at IPNT in Nha Trang. For each egg shipment, one egg strip was randomly selected and a sample of 100 eggs were removed. Eggs were assessed visually as intact, collapsed or hatched, and were counted. Eggs were then transferred to a hatching solution containing a small amount of larval diet as described below. After 24 hours the numbers of hatched larvae were counted and the hatch rate was calculated against the number of intact eggs above.

Eggs shipped from NIHE were hatched and reared in the IPNT insectary, where temperatures ranged between 26–31°C. For the Tri Nguyen releases, larvae (400/bucket) were reared in 4 L buckets and fed a diet of ground Tetramin Tropical Tablets (Tetra Holding [US] Inc. Germany, Product number 16110). When approximately 90% of larvae had pupated, 30 larvae/pupae were transferred into individual plastic cups (6 cm diameter x 10 cm high). A mesh cover was placed on each cup and adults were maintained for 3–4 days on 20% sucrose solution. Release cups were transferred to crates for transport to Tri Nguyen village via vehicle and boat. Release cups were maintained under ambient temperature conditions for 1.5 hours during transfer from the IPNT insectary to Tri Nguyen village.

For the Vinh Luong releases in 2018, a separate *w*Mel *Ae. aegypti* line was created in the NIHE insectary as above, with the exception that infected females were mated to uninfected males (F1) collected from two locations in Nha Trang City ( Table 1). After backcrossing (six generations) the colony was then maintained with the addition of Nha Trang City wild type (F1) males at a ratio of 10–20% per generation. Eggs were transferred via courier to the rearing facility at IPNT, where they were hatched and reared in trays (61 x 42 x 15 cm) containing 12 liters of tap water and were fed JBL NovoTab pellets (JBL, Neuhofen, Germany, Product number 302300). Larvae/pupae (100–120) were placed into individual plastic cups (850 mL) containing 250 mL of tap water. When approximately 90% of larvae had pupated, a mesh cover was placed on each cup and adults were maintained for 3–4 days on 20% sucrose solution. Release cups were transferred to crates for transport to the release site. Release cups were maintained under ambient temperature conditions for up to one hour during transfer from the IPNT insectary to Vinh Luong Ward.

Prior to releases in Vinh Luong, the *Wolbachia* mosquito line was characterized in terms of key fitness traits including adult female fecundity, egg hatch rate and *Wolbachia* maternal transmission efficiency as described above. Insecticide susceptibility was also assessed using previously described methods ( WHO, 2013). Insecticide type and concentrations ( Table 1) were in line with recommendations for *Ae. aegypti* mosquitoes and followed the WHO standard bioassay method ( WHO, 2013). Insecticide impregnated papers were purchased from the WHO Collaborating Centre at the Universiti Sains Malaysia, Penang, Malaysia. Each test included five replicate tubes for each insecticide and two negative control tubes, with 20 female *Ae. aegypti* mosquitoes per tube. The mosquitoes were three to five days old, fed with sugar only. Mosquitoes were kept in a paper-free tube for one hour to adapt, transferred to the tube containing insecticide-impregnated paper for one hour, then transferred back to the holding tube, with access to sugar solution, for 24 hours. Dead and live mosquitoes were counted after 24 hours.

### Releases

For Tri Nguyen releases, base maps showing the location of each household along with the 47 release zones that were used to coordinate field activities during the previous release of the *w*MelPop *Ae. aegypti* ( Nguyen *et al.*, 2015) were updated. Local community members were invited to join the project as project “collaborators”. Many of the collaborators had worked on the previous project that involved the release of *w*MelPop *Ae. aegypti.* These 47 collaborators were trained in mosquito release and monitoring activities and were then responsible for undertaking release and monitoring activities within their respective zones. During releases, cups of 3–4 day old adult mosquitoes (approx. 30 per cup) were transported by boat to the island each week. Each of the 47 project collaborators collected a box containing 9–30 release cups, and undertook releases in their respective neighborhood zones. Mosquitoes were released between 8:00 and 10:00 am, outside of each house that agreed to participate in the releases. Releases commenced on 14 May 2014 and were undertaken each week for 27 weeks.

For the Vinh Luong releases, release maps were created by overlaying a 50 x 50 m grid across the residential areas of the eight hamlets. During releases, cups containing 3–4 day old adult mosquitoes (approx. 100–120 per cup) were transported to the field via car, and then released by staff. One cup of mosquitoes was released inside each grid square each week (305 release grids). Mosquitoes were released between 07:30 and 10:30 hrs in shaded road-side locations. Releases commenced on 8 March 2018 and were undertaken each week, for 17 weeks.

### Community engagement

For the Tri Nguyen releases, communication and engagement activities followed the methods described in McNaughton & Duong (2014). This included sharing of information with community leaders and representatives from all households, via community events and meetings (33 events and meetings), door-knocking and one-on-one meetings with householders who were not engaged through community event or meeting (85 households), open letters to every household ( Ryan, 2021s), and community loudspeaker announcements (three announcements). A community reference group was established, with representation from six hamlet leaders to facilitate engagement with householders and identify any issues or concerns. The community collaborator system, utilized as part of the previous release involving *w*MelPop *Wolbachia* mosquitoes in 2013 ( Nguyen *et al.*, 2015), was also re-established. Each collaborator was responsible for 10–20 households and assisted with distributing newsletters, updating householders on progress and activities, and providing feedback to the community reference group. In addition, a school-based education campaign was undertaken with the local primary school, and involved a presentation to each class on dengue and *Wolbachia* and a drawing competition about the impact of dengue on the community. Local media were proactively engaged about the project activities, resulting in 20 media articles in local and national newspaper and television outlets.

Prior to releases residents were asked to provide written consent for the release of *Wolbachia* mosquitoes around their houses. Of the 715 registered households, 695 (97.2%) agreed to participate and gave consent for the release of mosquitoes outside their houses, 4 (0.6%) households did not agree for releases outside their houses, and 16 households did not complete the consent form.

For the Vinh Luong releases, communication and community engagement activities followed the Public Acceptance Model (PAM) as described in O'Neill *et al.* (2019). The community engagement activities were undertaken over a two-year period and involved the following:

1. Raising broad community and stakeholder awareness across Nha Trang City. Information was provided to residents and key stakeholders about *Wolbachia*, and mosquito releases and monitoring activities via various channels, including mass communication (the project’s website, community loudspeaker system, newspapers, TV, radio), school outreach programs, direct engagement with the local governments at different levels of administration, and community events using the existing community networks (heads and health collaborators of hamlets).

2. Quantitative surveys to assess community support in Vinh Luong. Three cross-sectional surveys were undertaken using a stratified random sampling method with the sample size of 370 participants (different participants for each of the surveys). Two pre-release surveys were undertaken prior to and after conducting communication and engagement activities. A follow-up post-release survey was undertaken three months after the start of mosquito releases. The initial pre-release survey prior to commencement of communication and engagement activities indicated high householder support and willingness to participate in releases (66.2%), or support for releases but not direct participation in releases (25.1%). A small proportion of householders were undecided whether they supported mosquito releases (6.5%), and only eight (2.2%) households indicated they did not support releases. After completion of the communication and engagement activities, the pre-release survey indicated householder support and willingness to participate had increased (83.8%), and support for releases but not direct participation in releases was 13.2%. Only a small proportion of householders were undecided whether they supported mosquito releases (2.4%), and only two (0.5%) households indicated they did not support releases. Similar results were found in the post-release survey undertaken three months after commencement of releases (88.1% of households support and willing to participate; 7.0% of households support but not willing to participate; 4.1% undecided whether they support releases; three households [0.8%] indicated they did not support releases).

3. Establishment of an issues management system. The system enabled community members to easily contact the project with any questions and concerns and have them quickly addressed by project staff typically within 24 hours of receipt. The system also allowed residents to opt in or out of direct participation in release and monitoring activities.

4. Community reference group. A community reference group was established with representatives from government organizations and community unions in Nha Trang City. The reference group’s function was to independently review activities to ensure that engagement was carried out in accordance with our stated Public Participation Principles ( O’Neill *et al.*, 2019).

As a requirement for institutional review board (IRB) approval, informed consent for the release of *Wolbachia* mosquitoes was obtained from a subsample of households (n=370) in Vinh Luong. Participating households were the same as those that participated in the 2nd pre-release survey of community acceptance as described in 2) above. Participation of households was voluntary, with the head (or representative) of each household asked to provide individual consent to undertake *Wolbachia* mosquito releases in their community. Of the 370 households, 100% completed the consent form, with all households providing consent for the release of mosquitoes in their community. In addition, 10 community meetings were held with representatives from households from each hamlet (total of 828 household participants), with verbal approval for releases from 100% of participants. Final written approval for releases was provided by the local authority, after reviewing the results from community engagement, and feedback from the community reference group.

### Field monitoring

Adult mosquito collections were undertaken during and after releases using BG Sentinel (BGS) traps (Biogents AG, Regensburg, Germany, Product number NR10030). The number and density of BGS traps in each area varied over time. In Tri Nguyen, initially 45–50 BGS traps were distributed throughout the release area (approximately two BGS traps per ha). After 41 months, this was reduced to 20 BGS traps (approximately one BGS trap per ha). In Vinh Luong, initially 42 BGS traps were distributed throughout the release area (approximately one BGS trap per 2.5 ha). After 15 months this was reduced to 15–20 BGS traps (approximately one BGS trap per 5 ha). Mosquitoes were collected from the BGS traps every 1–2 weeks and returned to the laboratory for sorting, morphological identification and counting. *Aedes aegypti* samples were stored in 70% ethanol prior to screening for *Wolbachia* infection status. After completion of releases, BGS trap sampling was undertaken every one to four weeks for 33 months in Tri Nguyen, and for 18 months in Vinh Luong, after which time BGS trap sampling was undertaken periodically at 6–12 months intervals. During 2020, BGS trap sampling was disrupted due to social distancing requirements in response to COVID-19, with sampling recommencing in Vinh Luong in November 2020, and in Tri Nguyen in April 2021.

### *Wolbachia* maternal transmission assessments on field collected material from Tri Nguyen

Two separate collections were undertaken, the first in May 2015 involving surveys at 48 households, with collections from the four most common container types found in Tri Nguyen ( Knox *et al.*, 2007): concrete rainwater tanks (> 500 L), plastic 200 L drums, ceramic 100 L jars and vases (< 1 L) used for religious purposes (i.e. ancestral shrines in homes). The second survey was undertaken in May 2016 with collections from 200 L drums only. Samples of IV instars and pupae were collected from containers using a 200 mm diameter sampling net (100 µm zoological plankton mesh).

IV instars/pupae were returned to the laboratory and each IV instar/pupae was placed into an individual container and allowed to emerge. Males were discarded and up to 10 individual virgin females from each container were placed into a small cage. Into each cage 10 *Wolbachia* uninfected Nha Trang City colony (F1) males were added and allowed to mate with virgin females over a 24 h period. Mosquitoes were blood fed and the progeny were processed as described previously, except immatures were fed on JBL NovoTab fish food (JBL NovoTab, Neuhofen, Germany, Product number 302300).

### Container surveys in Tri Nguyen

To determine whether there was any association between the abundance of different container types and the prevalence of *Wolbachia* across Tri Nguyen, a container survey was undertaken in November 2015. Houses were selected from a list of 715 registered households in Tri Nguyen village, with selection of every fifth house. A total of 143 houses were selected, representing 20% of registered houses across the north (n=62), center (n=47) and south (n=40) areas. Samples of late instars and pupae were collected from all water holding containers using a 200 mm diameter sampling net (100 µm zoological plankton mesh) ( Knox *et al.*, 2007). Field container types were categorized according to the following: concrete rainwater tanks (> 500 L), plastic 200 L drums, ceramic 100 L jars, buckets (<10 L) and vases (< 1 L) used for religious purposes (i.e. ancestral shrines in homes), and other miscellaneous containers (discarded items, ant traps, etc). Up to 10 individuals were collected from each container and samples were then transferred to 70% ethanol prior to processing for *Wolbachia* infection using a Taqman qPCR assay ( Dar *et al.*, 2008; O’Neill *et al.*, 2019; Yeap *et al.*, 2014).

To determine whether there was any association between abiotic water characteristics in different container types and the prevalence of *Wolbachia* across Tri Nguyen, an abiotic survey of water quality in containers was undertaken in June 2016. The survey was undertaken across a transect of houses from the north (24 houses), center (13 houses) and south (13 houses) areas. Houses were randomly selected across from a north-south transect, and samples of late instars and pupae were collected from different types of containers as above. A water sample from each container was collected and tested for pH, salinity and conductivity using a handheld water quality meter (PCSTestr 35, Eutech Instruments, Singapore).

### Diagnostic screening of samples for *Wolbachia*

Colony, field collected mosquitoes from BGS traps, larval samples from field containers, and samples from maternal transmission were screened for *Wolbachia* using Taqman qPCR on a Roche LightCycler 480 using an internally controlled qualitative assay for the presence or absence of *Wolbachia* as previously described ( Dar *et al.*, 2008; O’Neill *et al.*, 2019; Yeap *et al.*, 2014). TM513 primers and probe were used for Tri Nguyen samples to keep consistency with primary results and WSP primers and probe was used for Vinh Luong ward to reduce the possible effect of gene copy number variation in comparison with WS0513 gene. The qPCR cycling program consisted of a denaturation at 95°C for 5 min followed by 45 cycles of PCR (denaturation at 95°C for 10 sec, annealing at 60°C for 15 sec, and extension at 72°C for 1 sec with single acquisition) followed by a cooling down step at 40°C for 10 sec. Using the data generated for the presence-absence of *Wolbachia*, the relative density of *Wolbachia* per cell was calculated using the Advanced Relative Quantification (∆∆C _T_- Method) function within the Roche LightCycler 480 Software.

### Weather data

Meteorological data including maximum, minimum and average daily temperature records and daily rainfall for Nha Trang City (Station ID 48877099999) were obtained from the National Centers for Environmental Information, National Oceanic and Atmospheric Administration ( Menne *et al.*, 2012a; Menne *et al.*, 2012b). Local temperature data was collected from inside houses in Tri Nguyen and Vinh Luong using temperature data loggers. Houses for hosting the temperature data loggers were selected from the list of BG sentinel houses based on their geographic coverage across the representative release areas. In Tri Nguyen, six data loggers (EasyLog EL-USB-2, Lascar Electronics, Kowloon, Hong Kong) were used to record hourly temperatures inside houses (two houses in each of north, center and south areas) between May 2014 and June 2017. From July 2017 to June 2019 the EasyLog EL-USB-2 data loggers above were replaced with 10 iButtons (iButton DS1923, Maxim Integrated, San Jose, CA USA). These were set to record hourly temperatures inside houses and were located in six houses in the north, one in the center, and three in the south. In Vinh Luong, 10 iButton (iButton DS1923, Maxim Integrated, San Jose, CA USA) temperature data loggers were used to record hourly temperatures in 10 houses from March 2018 to October 2019, and from November 2020 to April 2021.

### Ethical considerations and consent

The release of *Wolbachia* mosquitoes at Tri Nguyen, along with human blood feeding of mosquitoes, was approved by the institutional review board (IRB) of the National Institute of Hygiene and Epidemiology (Approval reference number: 32/HDD 15/12/2011) and then the IRB of Vietnam Ministry of Health (Approval reference number: 38/CN-BDGDD 04/04/2014). Volunteer bloodfeeders provided informed written consent (no children were involved). For releases, residents were asked to provide written consent for the release of *Wolbachia* mosquitoes around their houses. In Vinh Luong, the release of *Wolbachia* mosquitoes along with human blood feeding of mosquitoes, was approved by the IRBs of the National Institute of Hygiene and Epidemiology (Approval reference number: IRB-VN01057-19/2017 12/10/2017) and Vietnam Ministry of Health (Approval reference number: 151/CN-BDGDD 28/12/2017). In Vinh Luong, the head (or representative) of 370 randomly selected households was asked to provide written consent to undertake *Wolbachia* mosquito releases in their community.

### Statistical analysis

The number of *Ae. aegypti* mosquitoes caught over time was summarized as the mean count per BGS trap per week, in each of the three Tri Nguyen areas (north, central, south) and in Vinh Luong. Mixed-effects negative binomial regression was used to compare the number of *Ae. aegypti* caught per BGS trap per week, between pre- and post-release periods, with the inclusion of BGS trap as a random effect to account for clustering at the trap level. All analyses included a binary indicator for hot/cool season as a covariate (hot season = May - October; cool season November - April). The count ratio produced by the negative binomial regression model is the ratio of the mean number of *Ae. aegypti* caught per trap per week between areas or between release vs non-release periods. Data was analyzed by release status: pre-release, during release, and post-release, and between sites for the total observation period. Analysis of the total observation period was done with the inclusion of an indicator for release status as a covariate.

The density of *Wolbachia* in *Ae. aegypti* caught over time was summarized as the median relative density value in *Ae. aegypti* caught each week during the post-release period, in each of the three Tri Nguyen areas (north, central, south) and in Vinh Luong. A distributed lag linear regression model was used to fit weekly median *Wolbachia* density to the mean daily temperature measured inside houses. The model included values of weekly mean daily temperature lagged up to 5 weeks for the three Tri Nguyen areas and 4 weeks for Vinh Luong, with maximum lag determined using the Akaike Information Criterion (AIC). Newey-West standard errors were calculated to account for autocorrelation in weekly median *Wolbachia* density, with maximum lag 9 for the three Tri Nguyen areas and maximum lag 5 for Vinh Luong.

## Results and discussion

### Tri Nguyen

**Figure 4.**
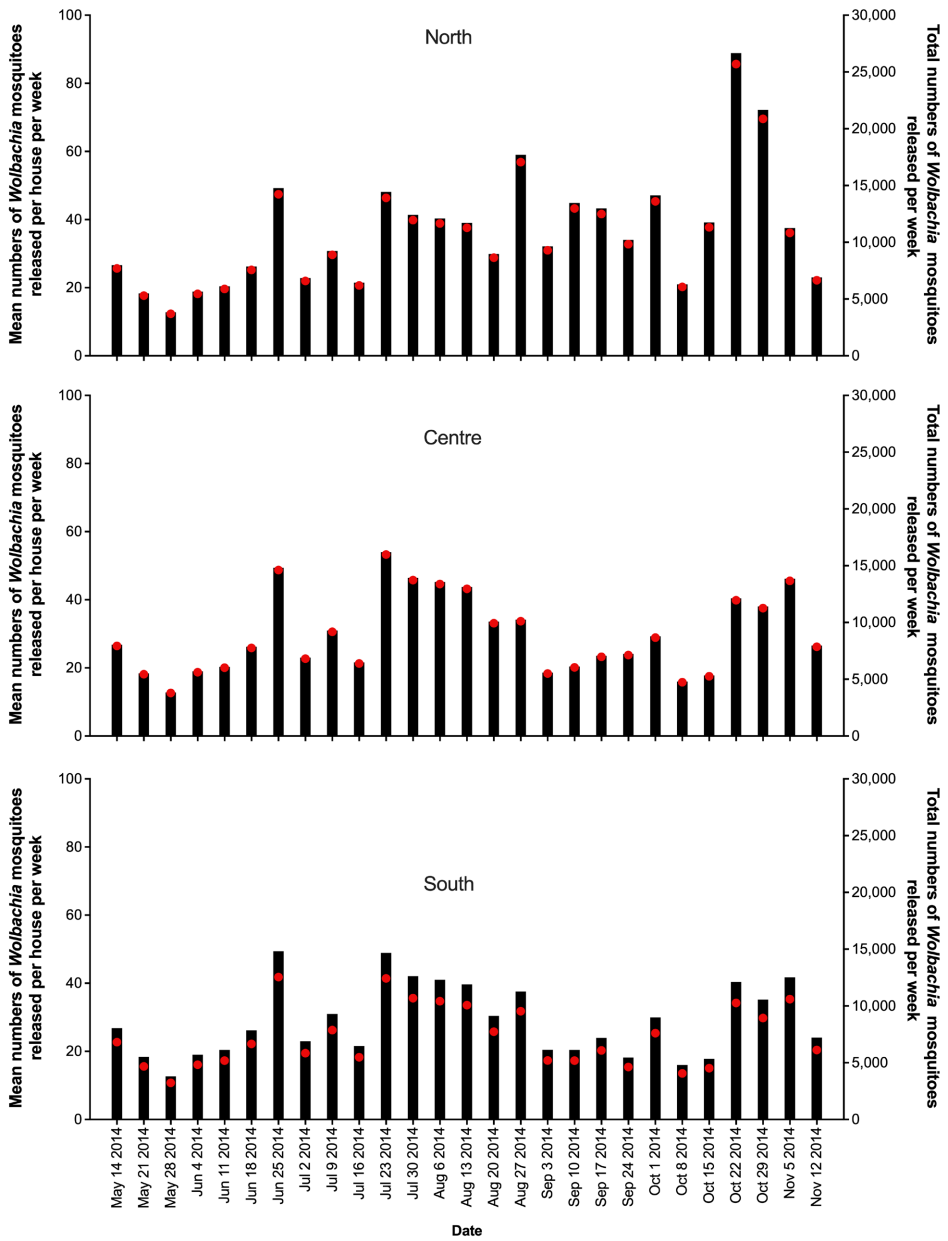
Mean numbers of *Wolbachia* mosquitoes released per house per week (black bars), and total numbers of *Wolbachia* mosquitoes released (red circles) in north, center and south areas in Tri Nguyen.

Releases of *w*Mel *Wolbachia Ae. aegypti* mosquitoes in Tri Nguyen were undertaken weekly for 27 weeks, with an average of 32.4 mosquitoes released per house per week (range 12.8 to 93.7 per house per week) ( Figure 4). In release weeks 8–9 (mid-July 2014) the *Wolbachia* prevalence in mosquitoes in BG traps in the north (34.1–38.3%) and central areas (28.7–41.9%) were low ( Figure 6), and release numbers were increased from week 11 (average 52.7 per house week) for 5–6 weeks in the central and south areas, and for the remainder of the releases in the north area ( Figure 4). By the end of 27 weeks of releases (14 November 2014) the *Wolbachia* infection prevalence in mosquitoes ranged from 77.3% to 86.6% across the three areas ( Figure 6, Figure 8). Over the next six months the *Wolbachia* infection prevalence in mosquitoes remained high and increased to 91.7–96.6% by mid-May 2015.

From June to December 2015, the *Wolbachia* infection prevalence in mosquitoes decreased to 22.5%, 26.3% and 55.4% in the north, central and south areas, respectively ( Figure 6, Figure 8). This period corresponded with the hot dry-season months from June to September, with average weekly temperatures in Nha Trang of 28.2–30.5°C, and average weekly maximum temperatures of 31.3–33.8°C. Monsoon rains occurred from October to December in Central Vietnam, and by December 2015 the weekly temperatures in Nha Trang had decreased to 26.3°C, and median weekly temperatures inside houses had decreased to 28°C. From January to May 2016, *Wolbachia* infection prevalence in mosquitoes in the north, central and south areas increased to 46.9, 40.9 and 94.1%, respectively ( Figure 6). During the following hot dry-season months from June to September 2016, *Wolbachia* infection prevalence decreased, and by the end of the monsoon rains in December 2016 *Wolbachia* infection prevalence was very low in the north (2.9%) and central areas (9.2%), yet remained high in the south area (75.0%) ( Figure 6, Figure 8).

From January 2017, *Wolbachia* infection prevalence in mosquitoes in the north was less than 10.1%, with no *Wolbachia* infection detected in mosquitoes except for mosquitoes from single trap collected in April 2021 ( Figure 6, Figure 9). In the central area, *Wolbachia* infection prevalence ranged between 6.6–58.3% between January 2017 to December 2018, but remained less than 5.1% from January 2019 onwards. In the south area, *Wolbachia* infection prevalence remained high with an average of 81.6% (range 46.8–97.7%). Infection levels below 20% are below the estimated unstable equilibrium point for *w*Mel in Cairns Australia (20–30%; Hoffmann *et al.*, 2011; Turelli & Barton, 2017); below this frequency threshold, *Wolbachia* infection frequency is expected to decline. The seasonal oscillations in *Wolbachia* infection frequencies in the south area (50–100%) did not approach the unstable equilibrium point, and this allowed for seasonal increases in *Wolbachia* infection frequencies in mosquitoes. In contrast, in the north and central areas, where the *Wolbachia* infection prevalence first declined to less than 20% in September 2016, the prevalence of *Wolbachia* generally declined thereafter ( Figure 6, Figure 8, Figure 9).

Overall, temperatures inside houses in Tri Nguyen were generally higher than the Nha Trang City meteorological data. Median weekly temperatures inside houses in Tri Nguyen were 1.7 +/- 0.6°C (s.d.) higher than the mean weekly temperatures in Nha Trang City, with median temperatures inside houses (31.0–32.5°C) during the hottest months approaching the mean weekly maximum temperatures in Nha Trang City.

To determine whether mosquitoes exposed to field conditions in Tri Nguyen had reduced maternal transmission of *Wolbachia* from infected females to their offspring, collections of late instars and pupae were made from different types of field containers and emergent adult females were assessed for efficiency of *Wolbachia* maternal transmission ( Table 2). In May 2015 when median weekly temperatures in houses were at their highest (30.5–32.5°C), imperfect *Wolbachia* maternal transmission was found across all three areas, with 53.6–69.0% of progeny from *Wolbachia*-infected females found to be positive for *Wolbachia*. There was no clear association between the type of container that females were collected from and maternal transmission. Repeat collections from drums in the north and south areas in May 2016, when median weekly temperatures were similarly high (31.5–32.5°C), found imperfect maternal transmission of *Wolbachia* from infected females, with only 16.7% and 60.5% of progeny from *Wolbachia* infected females found to be positive for *Wolbachia*, from the north and south areas, respectively.

**Table 2.** *Wolbachia* maternal transmission rates in female *Aedes aegypti* sourced as IV instars/pupae from different locations and container types in Tri Nguyen, May 2015 and May 2016.

Area	Container Type	Number of containers sampled	Number of containers maternal transmission assessed	Number progeny tested	Number progeny *Wolbachia* +ve	Maternal transmission	
						Percent	(Range ^1^)
*May 2015 Collection Period*							
North	Vase	3	2	34	34	100.0	(100.0-100.0)
	Jar	4	3	217	109	50.2	(0-100.0)
	Drum	4	3	274	194	70.8	(20.0-100.0)
	Tank	4	2	15	13	86.7	(60.0-100.0)
**Sub total**		**15**	**10**	**540**	**350**	**64.8**	
Center	Vase	5	2	78	36	46.2	(0-100.0)
	Jar	5	2	286	221	77.3	(0-100.0)
	Drum	5	4	74	53	71.6	(0-100.0)
	Tank	4	2	54	40	74.1	(0-100.0)
**Sub total**		**19**	**10**	**492**	**350**	**71.1**	
South	Vase	9	3	125	35	28.0	(0-85.0)
	Jar	3	2	80	62	77.5	(60.100.0)
	Drum	5	2	60	45	75.0	(40.0-85.0)
	Tank	1	0	0	0		
**Sub total**		**18**	**7**	**265**	**142**	**53.6**	
**Total**		**52**	**27**	**1,297**	**842**	**64.9**	
*May 2016 Collection Period*							
North	Drum	12	3	30	5	16.7	(0-50.0)
South	Drum	11	4	195	118	60.5	(0-100.0)
**Total**		**23**	**7**	**225**	**123**	**54.7**	

^1^ Percent maternal transmission per female

To investigate possible factors influencing the heterogeneity in *Wolbachia* infection prevalence in Tri Nguyen, container surveys were undertaken in November 2015 ( Table 3). Houses in the north area had the highest mean numbers of containers per house (13.0), compared with houses in the central (11.5) and south areas (8.2). The prevalence of *Ae. aegypti* immatures in containers was high in all areas, ranging from 17.2–23.1% of surveyed containers. *Wolbachia* infection prevalence in immatures from different container types ranged from 15.5–41.4% in the north, 22.5–35.5% in the center, and 56.7–92.9% in the south. Overall, the *Wolbachia* infection prevalence in immature stages collected from containers in the different areas matched those found in adult *Ae. aegypti* collected in BGS traps during November 2015 (north 26.1–38.8%; center 23.8–38.8%, south 58.7–76.9%). The *Wolbachia* infection prevalence in *Ae. aegypti* immatures, pooled at the house level, shows significant spatial heterogeneity in *Wolbachia* infection prevalence ( Figure 11). Container surveys in June 2016 found slightly elevated pH (7.93–7.96), salinity (1.4–2.0 ppt) and conductivity (2525–3512) levels in water in containers in the central and the south areas, compared to the north ( Table 4).

**Table 3.** Water container surveys in Tri Nguyen - prevalence of *Ae. aegypti* immatures and *Wolbachia* infection in III/IV instars and pupae collected from field containers in northern, central and southern areas in November 2015.

Area	# Houses surveyed	Container type	Mean cont. per house	Number cont. surveyed	Number cont. +ve *Ae. aegypti*	% cont. +ve *Ae. aegypti*	*Wolbachia* prevalence			
							Number cont. screened for *Wolbachia*	Number immatures screened for *Wolbachia*	Number immatures +ve *Wolbachia*	% *Wolbachia* infection in immatures
North	62	Tanks	2.4	146	26	17.8	26	225	53	23.6
		Drums	4.3	266	57	21.4	57	442	133	30.1
		Jars	0.6	35	13	37.1	13	122	41	33.6
		Buckets	3.3	206	20	9.7	20	142	22	15.5
		Vases	1.3	82	12	14.6	12	101	22	21.8
		Other	1.2	74	7	9.5	7	58	24	41.4
		**Subtotal**	**13.0**	**809**	**135**	**16.7**	**135**	**1,090**	**295**	**27.1**
Center	47	Tanks	2.1	99	22	28.2	28	233	46	19.7
		Drums	3.1	147	41	27.9	41	350	94	26.9
		Jars	1.3	63	25	39.7	25	228	81	35.5
		Buckets	2.9	138	21	15.2	21	171	44	25.7
		Vases	0.9	41	3	7.3	3	22	7	31.8
		Other	1.1	52	4	7.7	4	40	9	22.5
		**Subtotal**	**11.5**	**540**	**122**	**22.6**	**122**	**1044**	**281**	**26.9**
South	40	Tanks	2.3	93	19	20.4	18	160	110	68.8
		Drums	2.4	97	30	30.9	30	253	181	71.5
		Jars	0.6	25	12	48.0	12	92	74	80.4
		Buckets	1.5	59	9	15.3	9	67	38	56.7
		Vases	0.8	31	1	3.2	0	0	0	
		Other	0.6	24	2	8.3	2	14	13	92.9
		**Subtotal**	**8.2**	**329**	**72**	**21.9**	**71**	**586**	**416**	**71.0**

**Table 4.** Water container surveys in Tri Nguyen - prevalence of *Ae. aegypti* immatures and abiotic water parameters in water storage containers in northern, central and southern areas in June 2016.

Area	# Houses surveyed	Container type	Mean cont. per house	Number cont. surveyed	Number cont. +ve *Ae. aegypti*	% cont. +ve *Ae. * *aegypti*	*Water parameters*		
							pH	Salinity (ppt)	Conductivity
North	24	Tanks	2.4	58	6	10.3	7.93	0	178
		Drums	1.2	28	11	39.3	7.56	0	348
		Jars	1.0	24	6	25.0	7.48	1.1	513
		Buckets	1.1	26	7	26.9	7.16	0.1	521
		Vases	0.6	14	0	0	5.87	0	389
		Other	0.1	2	0	0	7.38	0	733
		**Subtotal**	**6.3**	**152**	**30**	**19.7**	**7.46**	**0.0**	**348**
Center	13	Tanks	2.6	34	3	8.8	8.20	1.1	2056
		Drums	2.6	34	11	32.4	7.84	1.6	2783
		Jars	0.8	10	6	60.0	7.77	1.2	2418
		Buckets	2.2	28	4	14.3	7.99	1.7	2928
		Vases	0.2	3	0	0.0	5.87	0	255
		Other	0.6	8	1	12.5	7.92	1.6	849
		**Subtotal**	**9.0**	**117**	**25**	**21.4**	**7.93**	**1.4**	**2515**
South	13	Tanks	1.2	16	2	12.5	8.06	2.5	4150
		Drums	2.5	33	9	27.3	7.99	1.7	2966
		Jars	0.8	10	4	40.0	8.29	2.4	4130
		Buckets	1.7	22	5	22.7	7.90	2.3	4050
		Vases	0.4	5	0	0	7.04	1.0	1713
		Other	0.2	2	0	0	8.15	1.7	2887
		**Subtotal**	**6.8**	**88**	**20**	**20**	**7.96**	**2.0**	**3512**

### Vinh Luong

**Figure 5.**
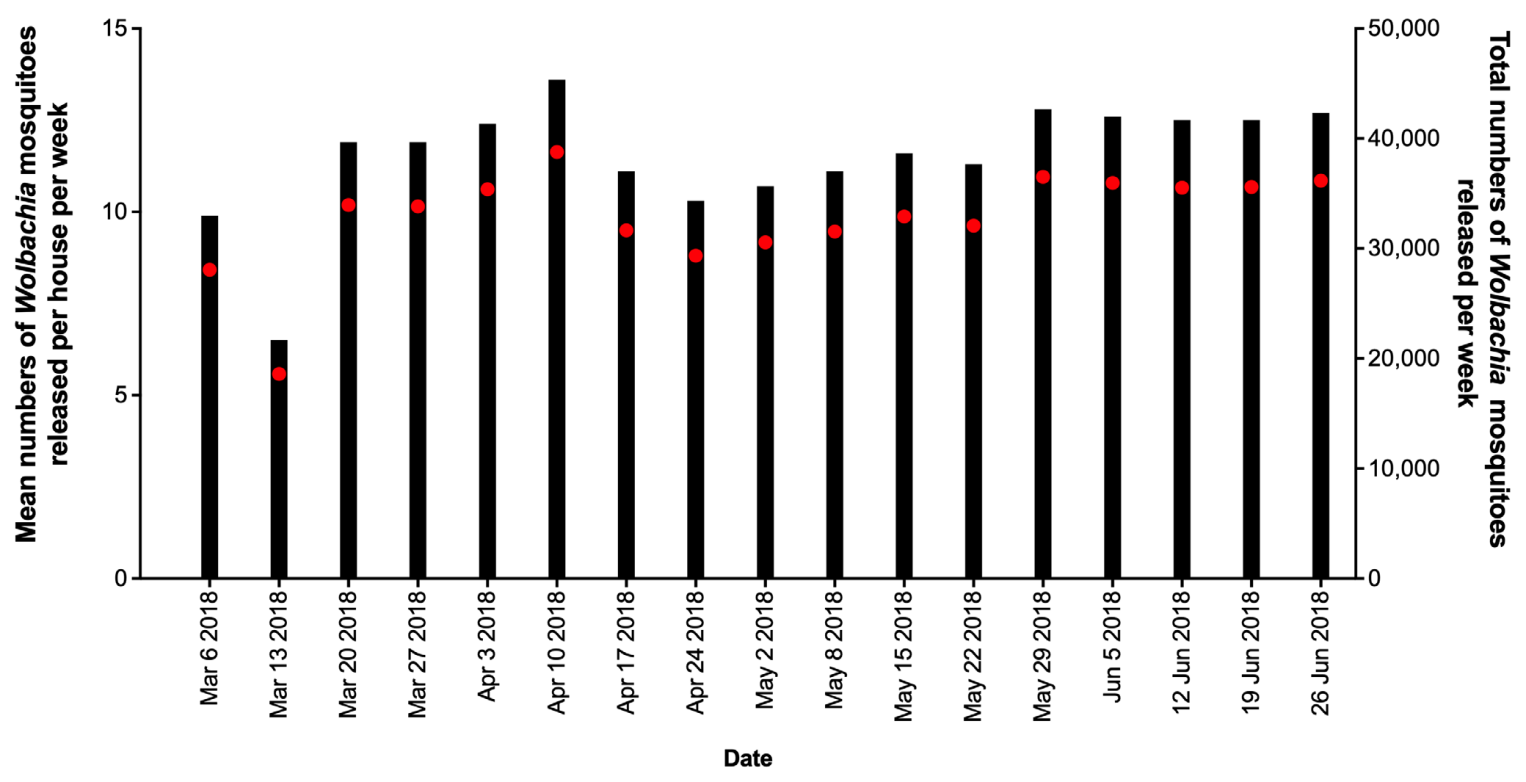
Mean numbers of *Wolbachia* mosquitoes released per house per week (black bars), and total numbers of *Wolbachia* mosquitoes released (red circles) in Vinh Luong per week.

Releases of *w*Mel *Wolbachia Ae. aegypti* mosquitoes in Vinh Luong were undertaken weekly for 17 weeks, with an average of 32,733 mosquitoes released per week (range 18,605 to 38,772) ( Figure 5). Compared with the Tri Nguyen releases where weekly releases were undertaken outside almost all houses (97.2%), the Vinh Luong releases were undertaken using evenly spaced 50 m x 50 m grids, with a single release inside each grid. This corresponded to a lower per house release density (one release point per nine houses in Vinh Luong, one release point per 1.03 houses in Tri Nguyen) and a lower weekly per house release rate (11.5 mosquitoes per house per week in Vinh Luong, 32.4 mosquitoes per house per week in Tri Nguyen). By the end of 17 weeks of releases (July 2018) the *Wolbachia* infection prevalence in mosquitoes was 78.9% ( Figure 7, Figure 10). Over the next four months the *Wolbachia* infection prevalence decreased to a low of 52.0% by October 2018, which coincided with high median weekly temperatures inside houses (29.1–32.1°C). The was followed by an increase in *Wolbachia* infection prevalence to 93.0% by March 2019, which coincided with lower seasonal temperatures (median weekly 25.6–29.1°C). Between June and October 2019, the *Wolbachia* infection prevalence decreased to 41.4%, which coincided with high median weekly temperatures inside houses (29.6–32.5°C). Field monitoring was interrupted from January to November 2020 due to social distancing requirements in response to COVID-19. *Wolbachia* infection prevalence in mosquitoes between November 2020 and March 2021 was high, ranging from 88.1–97.2%. ( Figure 7, Figure 10). Similar to Tri Nguyen, temperatures measured inside houses in Vinh Luong were warmer than the Nha Trang City meteorological data, with median temperatures inside houses 1.6 +/- 0.4 (s.d.) °C higher than the mean temperatures in Nha Trang City.

**Figure 6.**
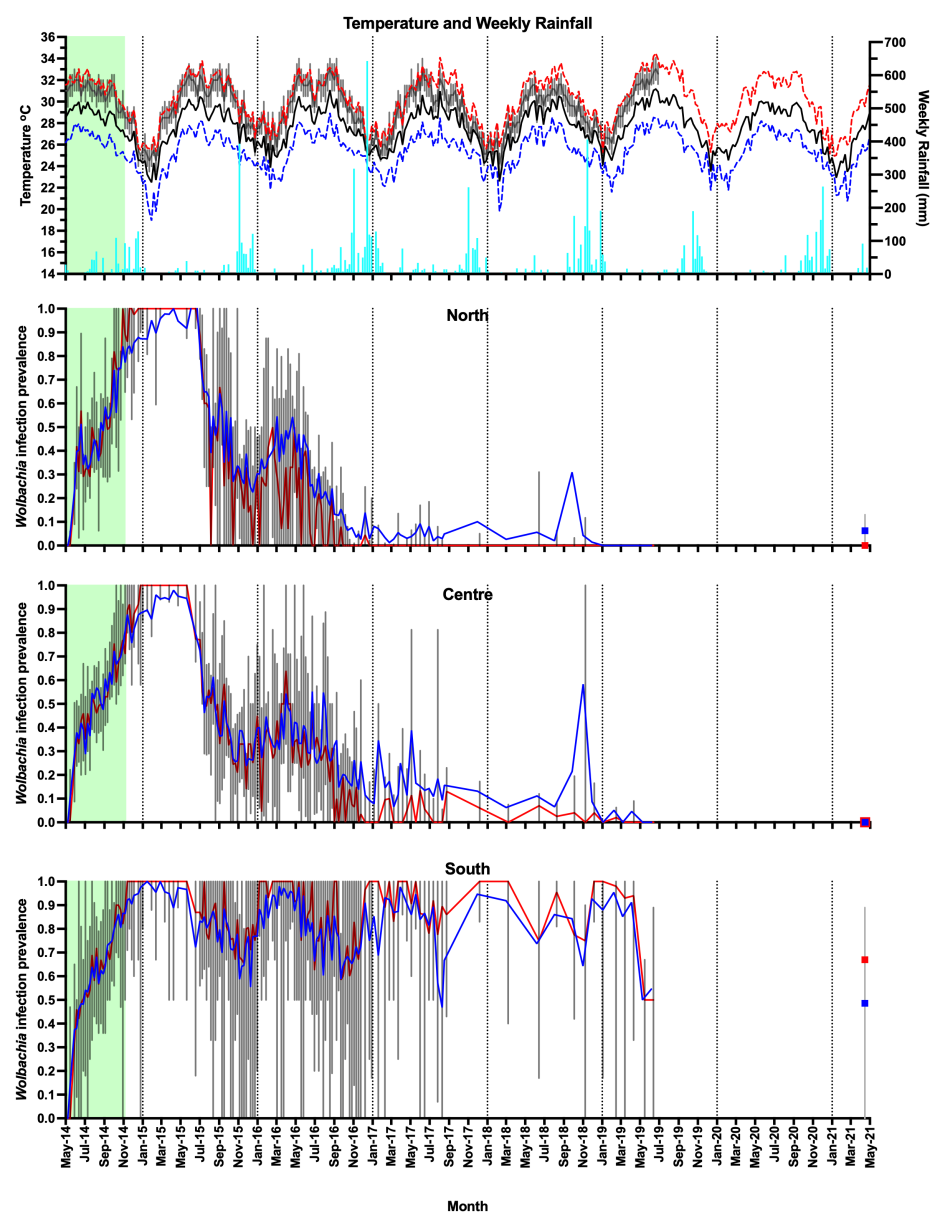
Weather station data for Nha Trang (mean daily maximum temperature per week - dashed red line, mean daily temperature per week - solid black line, mean daily minimum temperature per week - dashed dark blue line, weekly rainfall - light blue bars), household temperature data (median daily temperature per week - gray line, interquartile range - box plots), and *Wolbachia* infection prevalence in *Aedes aegypti* mosquitoes in Tri Nguyen ( *Wolbachia* prevalence in *Ae. aegypti* mosquitoes, total positives / total number tested - blue line, median *Wolbachia* prevalence in mosquitoes per BG trap collection - red line, interquartile range - box plots, no monitoring data between July 2019 and March 2021). Green shading represents *Wolbachia* mosquito release period (27 weeks).

**Figure 7.**
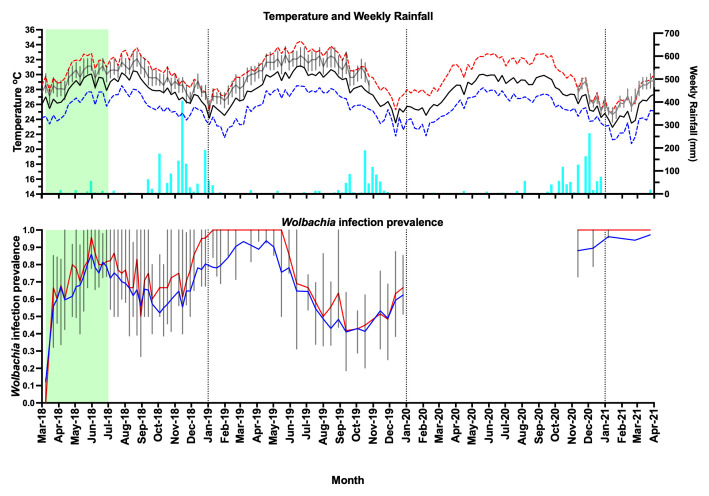
Weather station data for Nha Trang (mean daily maximum temperature per week - dashed red line, mean daily temperature per week - solid black line, mean daily minimum temperature per week - dashed dark blue line, weekly rainfall - light blue bars), household temperature data (median daily temperature per week - gray line, interquartile range - box plots), and *Wolbachia* infection prevalence in *Aedes aegypti* mosquitoes in Vinh Luong ( *Wolbachia* prevalence in *Ae. aegypti* mosquitoes, total positives / total number tested - blue line, median *Wolbachia* prevalence in mosquitoes per BG trap collection - red line, interquartile range - box plots, no monitoring data between January 2020 and October 2020). Green shading represents *Wolbachia* mosquito release period (17 weeks).

**Figure 8.**
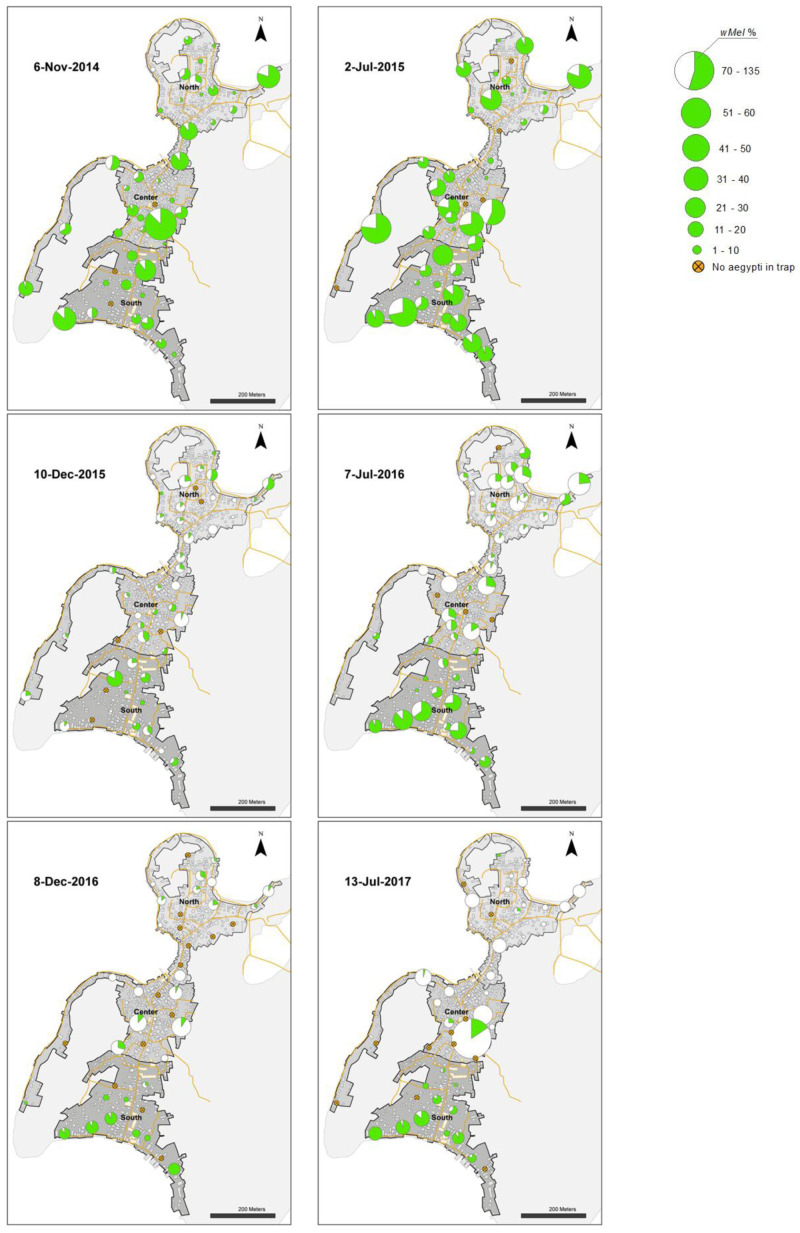
*Wolbachia* frequencies in *Aedes aegypti* mosquitoes collected in BG Sentinel traps in north, center and south areas in Tri Nguyen (6 November 2014 to 11 June 2018).

**Figure 9.**
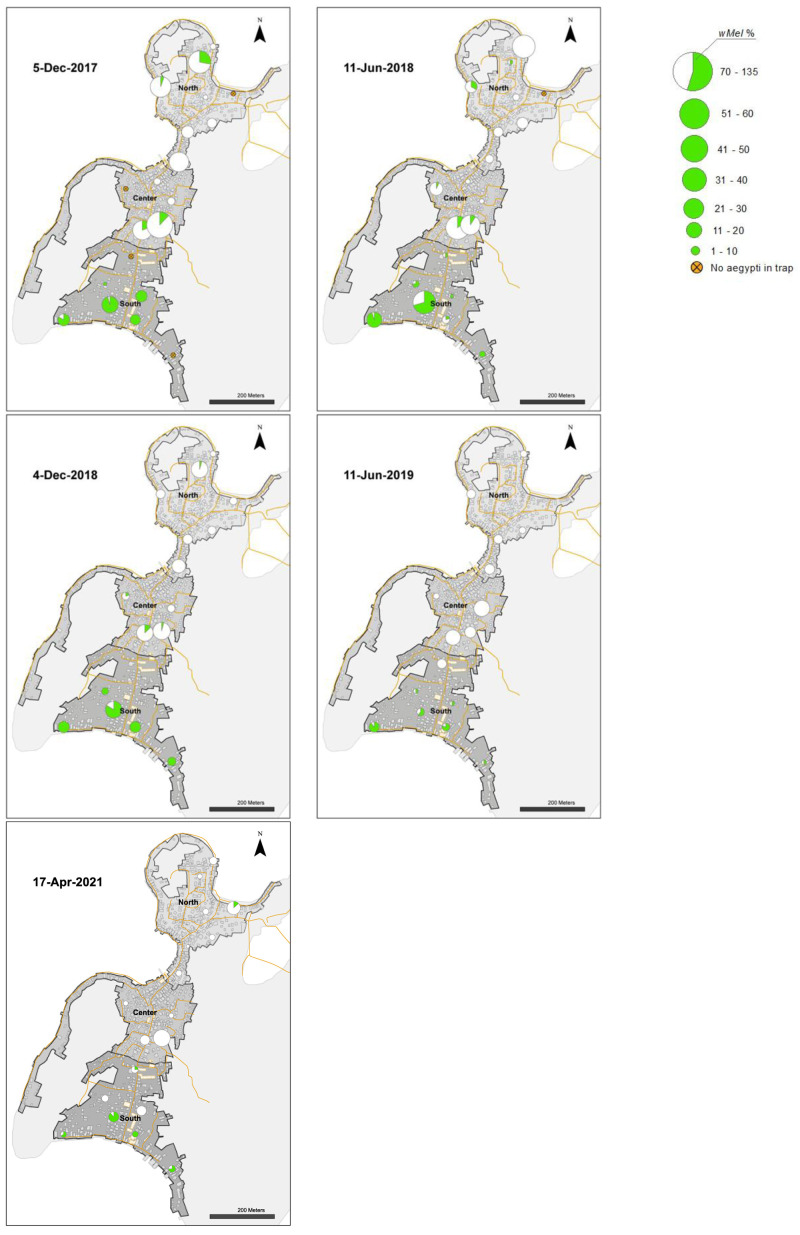
*Wolbachia* frequencies in *Aedes aegypti* mosquitoes collected in BG Sentinel traps in north, center and south areas in Tri Nguyen (4 December 2018 to 17 April 2021).

**Figure 10.**
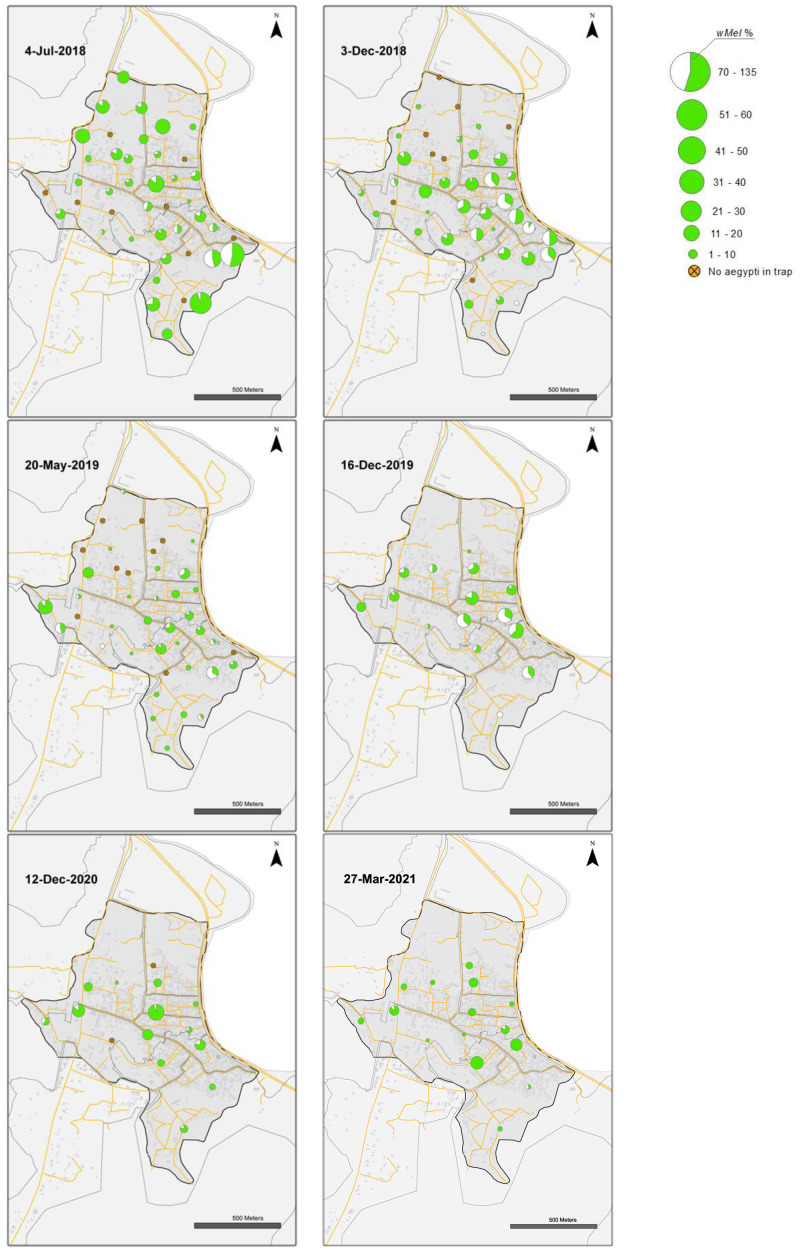
*Wolbachia* frequencies in *Aedes aegypti* mosquitoes collected in BG Sentinel traps in Vinh Luong (July 2018 to 27 March 2021).

**Figure 11.**
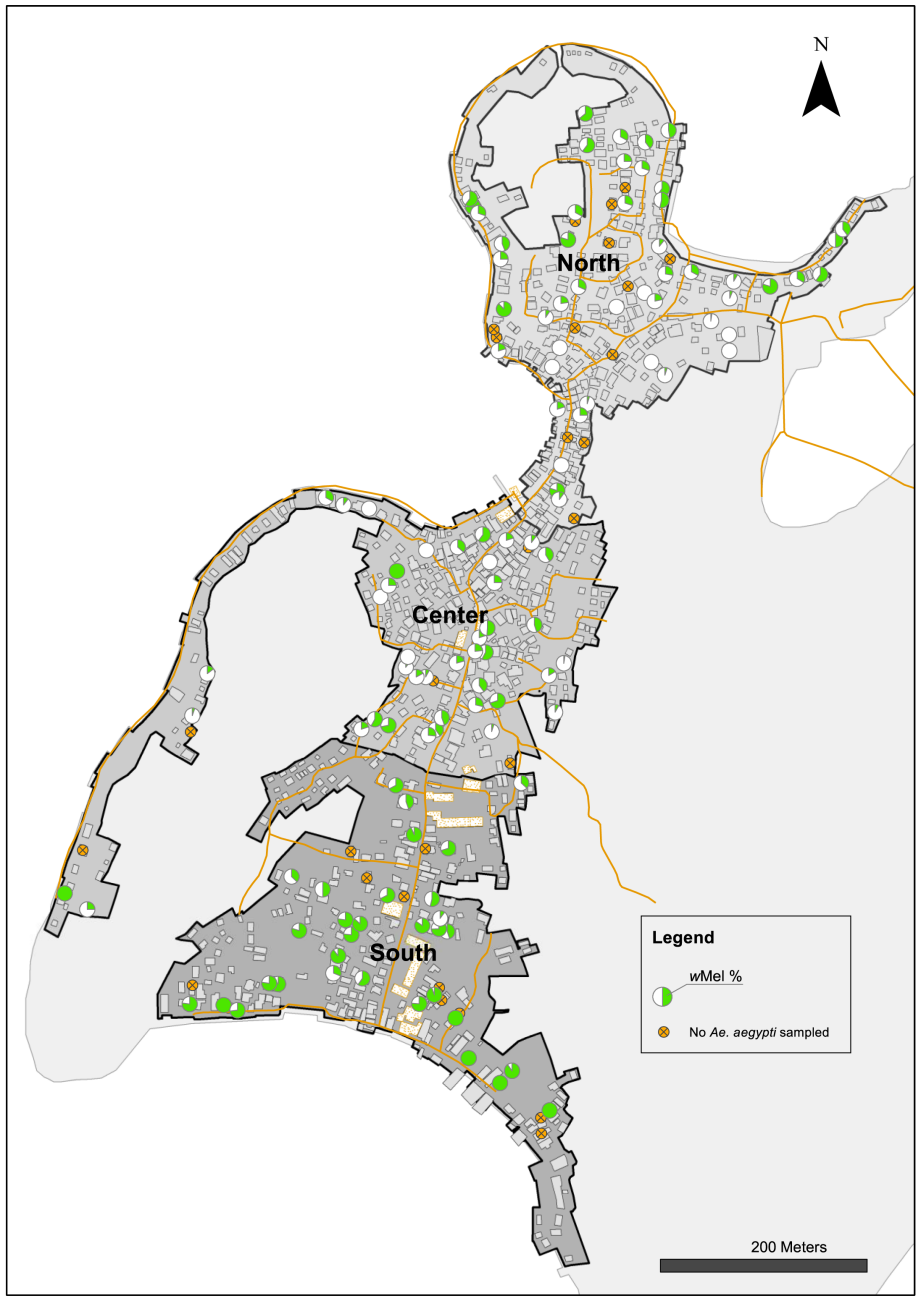
*Wolbachia* frequency in *Ae. aegypti* immatures collected from water holding containers in north (27.1%), center (26.9%) and south (71.0%) areas in Tri Nguyen in November 2015. *Wolbachia* frequency calculated based on pooled samples from all surveyed containers at each house (mean 11, range 1–27 surveyed containers per house).

### Effects of temperature on *Wolbachia* infection prevalence

The fluctuations in *w*Mel *Wolbachia* infection prevalence in mosquitoes in these two communities in Nha Trang in central Vietnam have consistent seasonal patterns, with reduced *Wolbachia* infection prevalence in mosquitoes during the hot dry seasons, followed by increased prevalence during the cooler seasons. This is consistent with recent laboratory and semi-field experiments investigating the effects of elevated temperatures on mosquito fitness and the stability of *Wolbachia* infection in *Ae. aegypti*. In the laboratory, immature stages (eggs and larvae) that were exposed to diurnal cycling temperatures that ranged from 30–40°C had lower *w*Mel *Wolbachia* densities in adult mosquitoes sampled at 0–2 days of age, compared with control mosquitoes reared at 20–30°C, and with partial recovery of *Wolbachia* levels in mosquitoes by 4–7 days of age ( Ulrich *et al.*, 2016). In a second laboratory study, exposure of *Ae. aegypti* larvae infected with different types of *Wolbachia* ( *w*Mel, *w*AlbB and *w*MelPop-CLA) to diurnal cyclical temperatures of 26–37°C resulted in reduced egg hatch in *w*Mel infected eggs, and reduced expression of cytoplasmic incompatibility and *Wolbachia* density in adult mosquitoes infected with the *w*Mel and *w*MelPop-CLA strains, but not the *w*AlbB strain ( Ross *et al.*, 2017). When immatures and adult females were reared and maintained at diurnal cyclical temperatures of 26–37°C, the *w*Mel and *w*MelPop-CLA infections were not transmitted to the next generation, which indicated a breakdown in maternal transmission fidelity. In contrast, the *w*AlbB *Wolbachia* infected line exhibited only partial breakdown in maternal transmission efficiency (88.5–91.7%) ( Ross *et al.*, 2017). Exposure of *Wolbachia* infected eggs to diurnal cycling temperatures of 30–40°C for seven days resulted in lower *w*Mel and *w*MelPop densities in adult females and males, compared with adults reared from eggs maintained at a constant 26°C ( Ross *et al.*, 2019). Further laboratory experiments indicated that exposure of larval stages to diurnal fluctuating temperatures between 26–37°C resulted in reduced *Wolbachia* density and starvation tolerance in the following generation, but only in female mosquitoes ( Foo *et al.*, 2019).

There is only limited previous data on the potential negative effects of temperature on *Wolbachia* establishment and stability under field conditions. In a semi-field study in northern Australia, *w*Mel *Wolbachia*-infected larvae were reared in containers placed in shaded and 50% shaded locations ( Ross *et al.*, 2019). The resulting adult males from the containers placed in 50% shaded locations, which reached temperatures of up to 39°C, were found to have partially lost their ability to induce cytoplasmic compatibility, and females had a greatly reduced egg hatch when crossed to infected males. The only relevant field experiment involving a field population of *Wolbachia*-infected *Ae. aegypti* was undertaken in Cairns, Australia, during a heatwave that occurred in November 2018, when temperatures reached 43.6°C ( Ross *et al.*, 2020). Eggs and immature stages (larvae and pupae) and adult mosquitoes were collected from ovitraps, field containers, sentinel containers that were placed in shaded and semi-shaded locations, and BGS sentinel traps throughout the central area of Cairns. In the month following the heatwave, *Wolbachia* infection prevalence was reduced to 83% in larvae sampled directly from field habitats and 88% in eggs collected from ovitraps, but recovered to be near 100% four months later ( Ross *et al.*, 2020). In this location, where *Wolbachia* had been established in local mosquito populations for more than five years ( Ryan *et al.*, 2020), high temperatures were found to have only temporary effects on *Wolbachia* frequencies.

The effects of elevated temperatures on the *Wolbachia*-induced viral blocking have been purportedly based on reduced *Wolbachia* densities in adult mosquitoes exposed to elevated rearing temperatures ( Ross *et al.*, 2017; Ross *et al.*, 2019; Ross *et al.*, 2020; Ulrich *et al.*, 2016); however, there is only limited data showing any direct effects of elevated temperatures on viral blocking. Laboratory experiments indicated that there was no significant effect on dengue 3 virus vector competence of *w*Mel *Wolbachia Ae. aegypti* mosquitoes reared at a constant 25°C, then exposed and maintained under two diurnal temperature settings with mean of 25°C and 28°C and a fluctuating range of 8°C (+/- 4 °C) ( Ye *et al.*, 2016). The two diurnal temperature regimes were found to significantly alter *Wolbachia* density in mosquitoes, with lower *Wolbachia* densities found in mosquitoes reared at the higher temperature regime; however, there was no association with dengue infection or the extrinsic incubation period of the pathogen in the mosquito ( Ye *et al.*, 2016). In contrast, exposure of *Ae. aegypti* immatures to higher cyclical temperatures of 28–36°C in the laboratory resulted in reduced *Wolbachia* density and levels of dengue 2 virus blocking in *w*Mel infected *Ae. aegypti*, compared with mosquitoes reared at a constant temperature of 27°C ( Mancini *et al.*, 2021). *w*Mel infected females reared at the higher temperature regime had significantly higher virus infection rates in heads and thoraces (33%) compared to *w*Mel females reared under constant temperature conditions (4.2%). No significant differences were found in dengue infection rates in *w*AlbB infected mosquitoes exposed to high-temperature and constant 27°C rearing conditions ( Mancini *et al.*, 2021). One of the few vector competence studies that have utilized field collected *Wolbachia Ae. aegypti* mosquitoes was undertaken in Tri Nguyen between March 2015 and June 2017 ( Carrington *et al.*, 2018). The dengue virus blocking ability of *w*Mel-infected *Ae. aegypti* was compared between laboratory reared wild-type and *w*Mel-infected *Ae. aegypti*, and field derived *w*Mel-infected *Ae. aegypti* collected from Tri Nguyen village. Compared with wild-type mosquitoes, the relative strength of dengue blocking in *w*Mel mosquitoes from Tri Nguyen was significantly greater in field-reared mosquitoes (mean reduction 85.9% +/- 6.3 SE) versus laboratory reared mosquitoes (67.9% +/- 5.2 SE, P = 0.033). The majority (30/48, 62.5%) of field collections for these studies were undertaken between March and October 2015, and coincided with fluctuations in *w*Mel infection prevalence in mosquitoes in the north, central and south areas of 26.9–100.0%, 26.0–98.0% and 69.9–100.0%, respectively. Despite these fluctuations in *w*Mel infection prevalence and sampling of mosquitoes from containers that were likely to be exposed to relatively high temperatures (median weekly temperatures inside houses ranged from 28.8–32.6°C), there was no evidence of any attenuation of virus blocking effects in *w*Mel infected *Ae. aegypti* mosquitoes collected from Tri Nguyen village.

The reduced fidelity in maternal transmission observed in female mosquitoes collected from Tri Nguyen Island in both May 2015 (64.1%) and May 2016 (54.7%) was consistent with the above laboratory studies that show incomplete maternal transmission when immature stages (eggs, larvae, pupae) are exposed to high, fluctuating diurnal temperatures ranging from 26–37°C. The high median temperatures measured inside houses in Tri Nguyen village (30.5–32.5°C) were probably similar to the temperatures found in the container habitats, and are therefore similar to the mean temperature of 31°C used in the fluctuating diurnal laboratory studies. Despite a complete breakdown in *Wolbachia* maternal transmission in *Ae. aegypti* in the laboratory when all life stages were held at 26–37°C, maternal transmission rates in Tri Nguyen village remained above 50%. This may reflect the heterogeneity in microclimates in individual containers and the different temperatures that immatures are exposed to. We also note the substantially warmer temperatures inside houses in both Tri Nguyen and Vinh Luong, compared to average temperatures reported from the Nha Trang weather station. These differences in temperatures between those measured inside houses and the weather station data, combined with differences in microclimate data experienced by immatures in different container types, means that caution should be used in drawing conclusions between weather station data and mosquito fitness and *Wolbachia* infection stability.

Although we did not measure the temperatures in water containers in the field, our study of maternal transmission in field containers set during the two consecutive hot seasons confirm the loss of *w*Mel infection in the progeny of *Wolbachia* infected female mosquitoes. This seasonal loss of *w*Mel infection in *Ae. aegypti* mosquitoes may be due to high temperatures in these larval habitats. Several studies have demonstrated that temperatures >35°C significantly reduce *Wolbachia* infections in *Ae. aegypti* larvae, especially younger instars ( Ross *et al.*, 2017; Ross *et al.*, 2019; Ross *et al.*, 2020; Ulrich *et al.*, 2016). Detailed entomological surveys on Tri Nguyen found that large water storage tanks and jars were responsible for over 90% of larval and pupal production ( Jeffery *et al.*, 2009). While temperatures in nearby Nha Trang indicate air temperatures in summer only occasionally exceed 35°C, it is likely that tanks and jars exposed to sunshine would be subject to solar gain and water within them could heat up. Heating of water in containers exposed to even partial sunshine can result in spikes in temperature > 35°C, and concurrent significant loss in *w*Mel density ( Ross *et al.*, 2019). Indeed, the amount of water inside tanks and jars may be at a minimum just when temperatures peak in the late dry season. Water in these tanks may heat well beyond 35°C and cure *w*Mel infections. Thus, it is critical that seasonal temperatures and *w*Mel levels in *Ae. aegypti* be monitored in a range of containers in shade and sunlight during the dry season.

**Figure 12.**
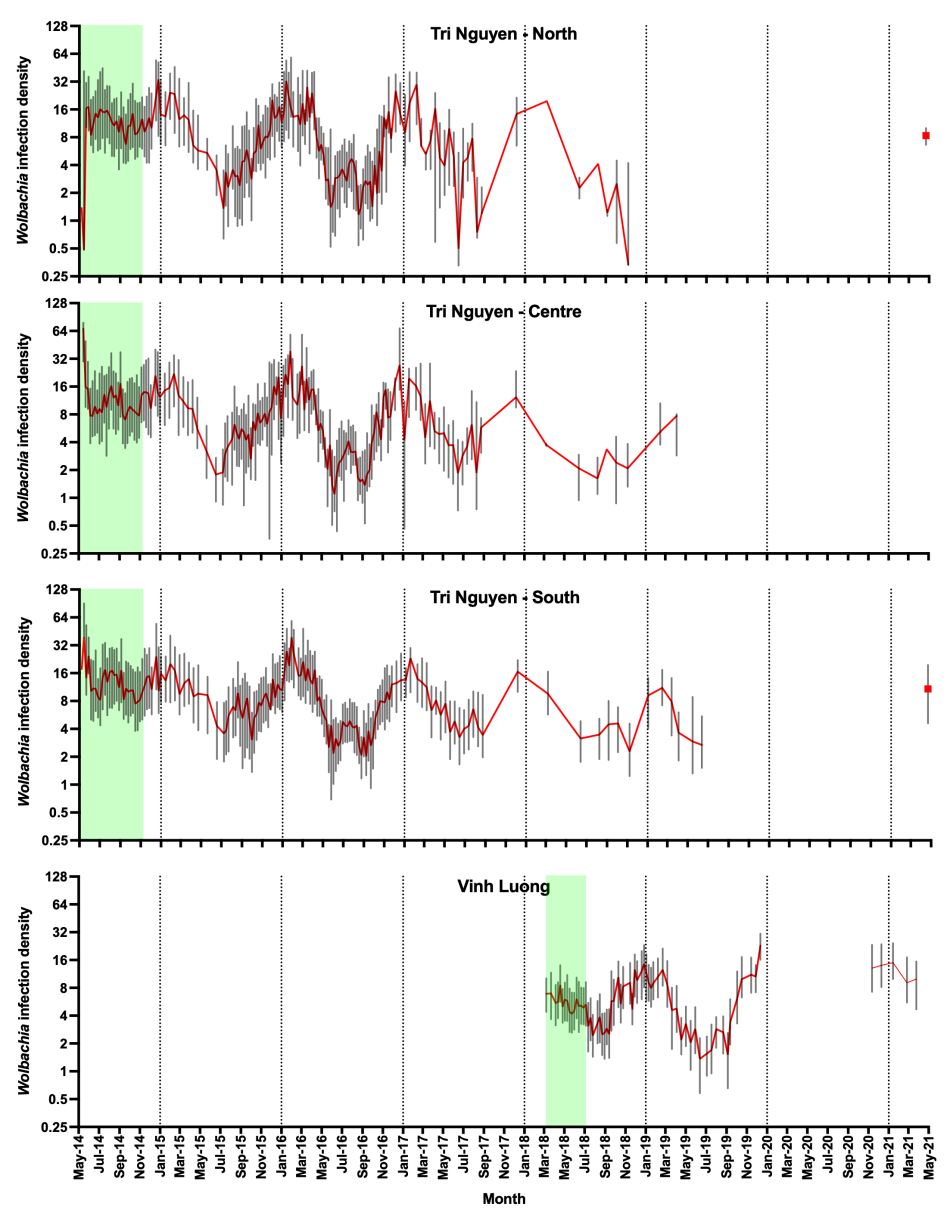
Median (+/- interquartile range) *Wolbachia* density in *Aedes aegypti* mosquitoes collected in BGS traps per week in north, centre and south zones in Tri Nguyen (no monitoring data between July 2019 and March 2021) and in Vinh Luong (no monitoring data between January 2020 and October 2020. Green shading represents *Wolbachia* mosquito release periods.

*Wolbachia* density in *Ae. aegypti* mosquitoes collected in BGS traps varied seasonally across both Tri Nguyen and Vinh Luong ( Figure 12). There was a significant negative correlation between the weekly median *Wolbachia* density in mosquitoes trapped during the post-release period and the weekly mean daily temperatures measured in households, in each region of Tri Nguyen and in Vinh Luong ( Table 5). This association was significant for both male and female *Wolbachia*-infected mosquitoes, but was much more pronounced in females. Associations were strongest when lagged temperature values of up to 5 weeks were included in the model for Tri Nguyen and up to 4 weeks for Vinh Luong.

**Table 5.** Correlation between median *w*Mel density in weekly collections of *Ae. aegypti* and the current and lagged mean daily temperature measured in households in Tri Nguyen and Vinh Luong. The coefficient is the linear combination of the regression coefficients (and their 95% confidence intervals) for the current and four prior weekly values of mean daily temperatures, interpretable as the (lagged) change in median relative *Wolbachia* density associated with a one degree increase in mean daily temperature. Standard errors are adjusted for autocorrelation in *w*Mel density.

Area	Coefficient (95% CI)
Tri Nguyen (North) Females Males	-5.18 (-7.16, -3.20) -1.24 (-1.79, -0.70)
Tri Nguyen (Center) Females Males	-4.26 (-6.30, -2.23) -1.21 (-1.86, -0.55)
Tri Nguyen (South) Females Males	-3.81 (-5.71, -1.91) -1.03 (-1.55, -0.51)
Vinh Luong Females Males	-2.24 (-2.62, -1.87) -0.73 (-0.86, -0.60)

### Other factors affecting *Wolbachia* infection prevalence

**Figure 13.**
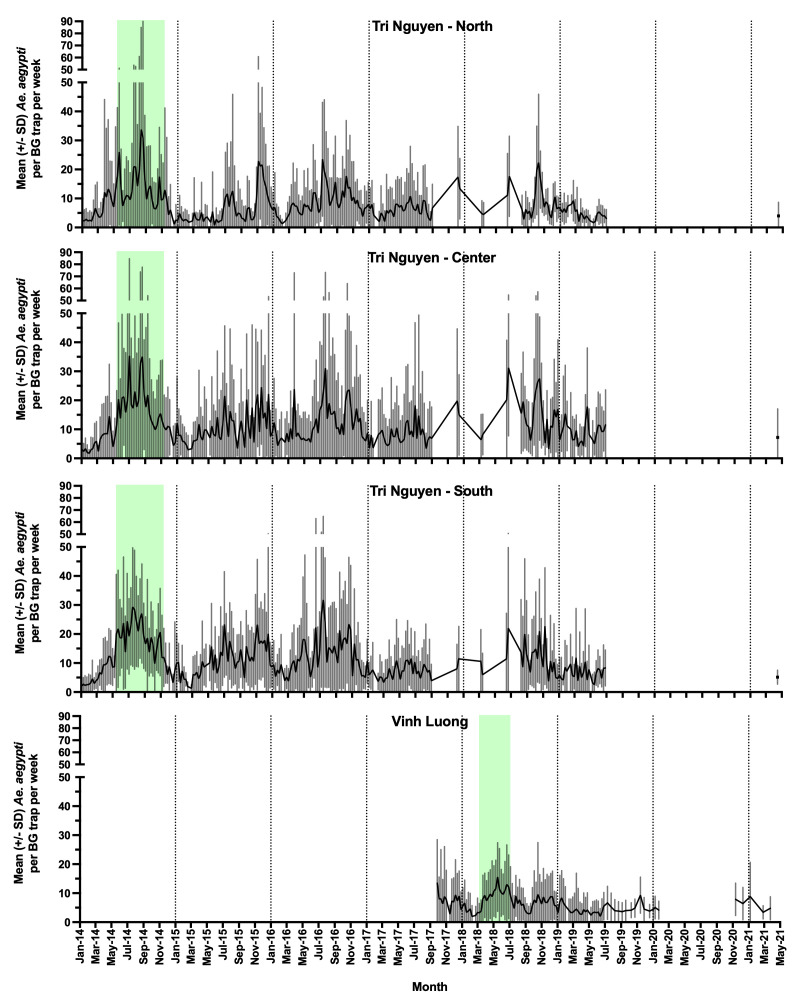
Mean (+/- standard deviation [SD]) numbers of *Aedes aegypti* mosquitoes collected in BGS traps per week in north, centre and south zones in Tri Nguyen (no monitoring data between July 2019 and March 2021) and in Vinh Luong (no monitoring data between January 2020 and October 2020). Green shading represents *Wolbachia* mosquito release periods.

There was no clear association between *Wolbachia* establishment in local mosquito populations and seasonal or spatial differences in *Ae. aegypti* abundance in BGS traps. *Ae. aegypti* adult mosquito numbers in BGS traps were significantly (P<0.05) higher during the hot season (May to October) compared with the cool season (November to April) across all sites (Tri Nguyen North *Ae. aegypti* mean catch ratio 1.28 [95% CI 1.18, 1.39]; Center 1.17 [1.07, 1.28]; South 1.42 [1.30, 1.54], Vinh Luong 1.22 [1.22, 1.32]) ( Figure 13). In terms of overall mean *Ae. aegypti* numbers in BGS traps during non-release periods, mosquito densities were significantly higher in Tri Nguyen in the South (Mean catch ratio 1.94 [95%CI 1.26, 2.98]) and Center zones (1.69 [1.16, 2.47]) compared with Vinh Luong; however the numbers in Tri Nguyen North were not significantly different to Vinh Luong (1.18 [0.84, 1.68]). Despite the similar and relatively low mosquito densities in Tri Nguyen in the North and in Vinh Luong, and similar seasonal variations in mosquito densities, *Wolbachia* persistence was high across Vinh Luong; whereas its persistence was low in the Tri Nguyen North zone. In contrast, the Tri Nguyen South zone had almost twice the mean numbers of *Ae. aegypti* in BGS traps, compared with Vinh Luong, and *Wolbachia* persistence was high across both sites. Comparisons of *Ae. aegypti* numbers in BGS traps before and after releases indicated that *Ae. aegypti* numbers in Tri Nguyen were higher in the post-release period compared with the pre-release period (Mean catch ratio 1.29 [95% CI 1.18, 1.41]), whereas in Vinh Luong the reverse was observed, with a reduction in numbers during the post-release period (Mean catch ratio 0.87 [95% CI 0.80, 0.96]). Within zone differences in *Ae. aegypti* numbers before and after releases were found in Tri Nguyen, with significantly higher numbers during the post release periods in the Center and South areas ( *Ae. aegypti* mean catch ratios 1.60 [95% CI 1.37, 1.88] and 1.33 [1.15, 1.55], respectively), but not in the North zone (1.04 [0.89, 1.22]). Although these analyses were adjusted for season, they may still be confounded by seasonality as the pre-release monitoring period in both locations was very short (4–5 months). Not surprisingly, the mean numbers of *Ae. aegypti* in BGS traps during the releases were higher than the numbers during non-release periods in both Tri Nguyen (Mean catch ratio 1.82 [95% CI 1.69, 1.96]) and Vinh Luong (1.98 [1.82, 2.15]).

The small-scale spatial heterogeneity in *Wolbachia* infection prevalence throughout Tri Nguyen, over distances of 200–300 m, suggests that other factors, besides ambient temperature, are important to *Wolbachia* persistence in this setting. The prevalence of containers was similar across the Tri Nguyen site, and, despite some variation in abiotic water parameters, we found no association between these container characteristics and *Wolbachia* infection prevalence. The persistence of *Wolbachia* in mosquito populations in the south area of Tri Nguyen village, for over seven years, may present an opportunity for direct selection of *Wolbachia* variants with higher thermal tolerance. Although laboratory experiments found limited capacity for *w*Mel infected *Ae. aegypti* to adapt to high temperatures ( Ross & Hoffmann, 2018), long term monitoring of the Tri Nguyen village site will determine whether *Wolbachia* can adapt to exposure to high temperatures and other potential environmental factors. The long-term persistence of *Wolbachia* in Vinh Luong, despite some seasonal fluctuations in *Wolbachia* infection prevalence, indicates that different underlying factors are associated with *Wolbachia* maintenance.

Given the successful establishment and persistence of *w*Mel *Wolbachia* in mosquito populations across a range of settings ( Garcia *et al.*, 2019; Gesto *et al.*, 2021; Hoffmann *et al.*, 2011; Hoffmann *et al.*, 2014; Indriani *et al.*, 2020; O'Neill *et al.*, 2019; Ryan *et al.*, 2020; Schmidt *et al.*, 2017; Tantowijoyo *et al*., 2020; Utarini *et al.*, 2021), some of which experience short term fluctuations in temperatures analogous to those experienced in Tri Nguyen village and Vinh Luong, it is clear that ambient measured temperatures alone may be insufficient to predict the success or failure of *w*Mel deployments. Elements of the built environment, as yet undetermined, may have important effects on *Wolbachia* establishment and persistence. In some complex urban environments, it may prove slow and operationally challenging to achieve a homogeneous high level of *Wolbachia* establishment. But it has been demonstrated in Niterói, Brazil, that measurable reductions in dengue, chikungunya and Zika disease accrue even at a moderate prevalence of *w*Mel in local *Ae. aegypti* populations (40% to >80%) ( Pinto *et al*., 2021).

The potential effects of elevated seasonal temperatures on *Wolbachia* establishment may become more important in areas with arid and temperate climates that experience wider yearly temperature ranges and higher summer temperatures compared to tropical climates. These arid and temperate climate areas are typically found at latitudes between 20–35°north and south. In comparison, areas with tropical climates (particularly tropical rainforest and tropical monsoon climates) are characterized by monthly temperatures above 18°C year-round (typically between 21–30°C), and the annual temperature range is normally very small. The global population at risk of dengue in 2015 was estimated to be 3.83 billion (roughly 53% of the global population) ( Messina *et al.*, 2019). Based on 2010 population estimates for various climate zones, approximately 2.2 billion people (58% of at the risk dengue population) reside in areas with tropical climates ( Center for International Earth Science Information Network, 2012). For most of these areas the environmental conditions are likely to be less extreme than those experienced in Tri Nguyen and Vinh Luong, and therefore *w*Mel releases are likely to result in stable establishment. Epidemiological modelling predicted that the establishment of *w*Mel globally, even with an intermediate efficacy (50% transmission reduction), would reduce global dengue incidence by up to 90% ( Cattarino *et al.*, 2020). If this was targeted in tropical areas where temperatures are amenable for *w*Mel, this would represent a potential overall reduction in global dengue burden of over 50%. For areas outside of the tropics, where the range of *Ae. aegypti* extends into areas that experience more extreme summer temperatures, *Wolbachia* strains that are more heat resistant such as *w*AlbB may be preferable ( Ross *et al*., 2017; Ross *et al.*, 2019). Currently, however, there is limited data on the field performance of the *w*AlbB strain. Small-scale releases of *w*AlbB mosquitoes in Malaysia resulted in heterogenous establishment, purported to be due to immigration of uninfected mosquitoes from surrounding areas ( Nazni *et al.*, 2019). However, *w*AlbB has been associated with negative fitness effects in *Ae. aegypti*, resulting in reduced fertility in adult females emerging from quiescent eggs exposed to moderate temperatures (20–30°C) ( Lau *et al.*, 2021). However, as shown in Tri Nguyen and Vinh Luong with *w*Mel, subtle fitness effects under different environmental conditions may lead to unknown consequences on *Wolbachia* spread and maintenance, and therefore it is important to better understand the field dynamics *Wolbachia* strains across different settings and across seasons.

## Data availability

### Underlying data

Figshare: VL Mosquito data. https://doi.org/10.6084/m9.figshare.15070803.v1 ( Ryan, 2021a).

Figshare: VL Release data. https://doi.org/10.6084/m9.figshare.15070797.v1 ( Ryan, 2021b).

Figshare: VL Temperature data - data logger. https://doi.org/10.6084/m9.figshare.15070794.v1 ( Ryan, 2021c).

Figshare: VL Temperature data - data logger raw. https://doi.org/10.6084/m9.figshare.15102129 ( Ryan, 2021d).

Figshare: TN Mosquito data. https://doi.org/10.6084/m9.figshare.15070785.v1 ( Ryan, 2021e).

Figshare: TN Release data. https://doi.org/10.6084/m9.figshare.15070782.v1 ( Ryan, 2021f).

Figshare: TN Temperature - data logger. https://doi.org/10.6084/m9.figshare.15070779.v1 ( Ryan, 2021g).

Figshare: TN Temperature - data logger raw. https://doi.org/10.6084/m9.figshare.15102150 ( Ryan, 2021h).

Figshare: VL Wolbachia colony infection rate. https://doi.org/10.6084/m9.figshare.15102204.v1 ( Ryan, 2021i).

Figshare: VL Insecticide resistance. https://doi.org/10.6084/m9.figshare.15102195.v1 ( Ryan, 2021j).

Figshare: VL Fecundity and hatch rate. https://doi.org/10.6084/m9.figshare.15102210.v1 ( Ryan, 2021k).

Figshare: VL Wolbachia colony maternal transmission. https://doi.org/10.6084/m9.figshare.15102198.v1 ( Ryan, 2021l).

Figshare: Field maternal transmission data. https://doi.org/10.6084/m9.figshare.15102192.v1 ( Ryan, 2021m).

Figshare: TN Water Quality Survey. https://doi.org/10.6084/m9.figshare.15102180.v1 ( Ryan, 2021n).

Figshare: TN Water container surveys wMel prevalence. https://doi.org/10.6084/m9.figshare.15102186.v1 ( Ryan, 2021o).

Figshare: VL Cross sectional surveys. https://doi.org/10.6084/m9.figshare.15102177 ( Ryan, 2021p).

Ryan, Peter (2021): TN BGS Ae. aegypti Abundance. figshare. Dataset. https://doi.org/10.6084/m9.figshare.17427434.v1

Ryan, Peter (2021): VL BGS Ae. aegypti Abundance. figshare. Dataset. https://doi.org/10.6084/m9.figshare.17427470.v1

Ryan, Peter (2021): Wolbachia density data. figshare. Dataset. https://doi.org/10.6084/m9.figshare.17427479.v1

### Extended data

Figshare: VL Communications Materials. https://doi.org/10.6084/m9.figshare.15185022 ( Ryan, 2021q).

Figshare: TN Communications Materials. https://doi.org/10.6084/m9.figshare.15184944 ( Ryan, 2021r).

Figshare: TN Open Letter. https://doi.org/10.6084/m9.figshare.15185241 ( Ryan, 2021s).

Data are available under the terms of the Creative Commons Attribution 4.0 International license (CC-BY 4.0).
