# Supplementary material for: Environmental factors influence the local establishment of Wolbachia in Aedes aegypti mosquitoes in two small communities in central Vietnam
Source: Gates Open Res. 2022 Apr 7;5:147. Originally published 2021 Sep 24. [Version 2] doi: 10.12688/gatesopenres.13347.2 (PMC9098883; doi:10.12688/gatesopenres.13347.2)
Supplement: Peer review history

# Peer review: Reviewer response for version 2

**DOI:** 10.21956/gatesopenres.14782.r31918

**Published:** 2022-05-12

**Authors:** Gonzalo M. Vazquez-Prokopec

**Affiliations:** 1 Department of Environmental Sciences, Emory University, Atlanta, GA, USA

All my comments were addressed.

Is the work clearly and accurately presented and does it cite the current literature?

Yes

If applicable, is the statistical analysis and its interpretation appropriate?

Yes

Are all the source data underlying the results available to ensure full reproducibility?

Yes

Is the study design appropriate and is the work technically sound?

Yes

Are the conclusions drawn adequately supported by the results?

Yes

Are sufficient details of methods and analysis provided to allow replication by others?

Yes

Reviewer Expertise:

Vector Ecology and Control

I confirm that I have read this submission and believe that I have an appropriate level of expertise to confirm that it is of an acceptable scientific standard.

# Peer review: Reviewer response for version 1

**DOI:** 10.21956/gatesopenres.14590.r31211

**Published:** 2021-11-18

**Authors:** Gonzalo M. Vazquez-Prokopec

**Affiliations:** 1 Department of Environmental Sciences, Emory University, Atlanta, GA, USA

There is mounting evidence showing that temperature and possibly other environmental and ecological conditions impact *Wolbachia* maternal transmission in transinfected *Aedes aegypti*. This study reports the findings from a large release of *Ae. aegypti* transinfected with the WMel strain of *Wolbachia* in Vietnam. In general, the manuscript is well written and provides important information regarding the limitations of *Wolbachia* population replacement in tropical urban environments.

 General comments:

1. While I agree that *Wolbachia* infection may be influenced by temperature, the study failed to demonstrate that temperature was the reason why *Wolbachia* prevalence was reduced and fragmented in each city. Given the warm season is also associated with high vector abundance, how are the authors certain that there was no effect of immigration of mosquitoes from neighboring areas, not receiving the intervention? Could it be that both immigration and temperature operated in this specific context?
2. While temperature effects on WMel are documented from the lab, this study did not conduct any experimental quantification of the role of temperature on WMel infection in the field. Again, it is speculative to assume temperature was the only cause.
3. While there is evidence of WMel infection presence, how about WMel density? Why are not loads of WMel in mosquitoes reported as in other WMP studies?

Minor comments:

1. The figures show mean numbers of mosquitoes but exclude the variability around the mean. Report standard errors or other measure.

Is the work clearly and accurately presented and does it cite the current literature?

Yes

If applicable, is the statistical analysis and its interpretation appropriate?

Yes

Are all the source data underlying the results available to ensure full reproducibility?

Yes

Is the study design appropriate and is the work technically sound?

Yes

Are the conclusions drawn adequately supported by the results?

Yes

Are sufficient details of methods and analysis provided to allow replication by others?

Yes

Reviewer Expertise:

Vector Ecology and Control

I confirm that I have read this submission and believe that I have an appropriate level of expertise to confirm that it is of an acceptable scientific standard, however I have significant reservations, as outlined above.

# Response

**Published:** 2021-12-28

**Authors:** Peter Ryan

**Affiliations:** World Mosquito Program, Vietnam

*While I agree that Wolbachia infection may be influenced by temperature, the study failed to demonstrate that temperature was the reason why Wolbachia prevalence was reduced and fragmented in each city. Given the warm season is also associated with high vector abundance, how are the authors certain that there was no effect of immigration of mosquitoes from neighboring areas, not receiving the intervention? Could it be that both immigration and temperature operated in this specific context?*

*Tri Nguyen village is on an island separated from the mainland and Wolbachia mosquito releases were undertaken throughout essentially all of the populated areas on the island. The number of immigrant mosquitoes on the island would be relatively small in relation to the native Ae. aegypti population. Similarly in Vinh Luong, while located on the mainland, it is essentially surrounded by uninhabited areas with limited habitat for Ae. aegypti, so immigration would be limited. We don't consider immigration of uninfected mosquitoes into the release areas to have played a major role in the observed fluctuations in Wolbachia.*

 In response to this reviewer's comment on seasonal variation in vector abundance and the effect on *Wolbachia* establishment, and also in response to Reviewer 1 comments on *Ae. aegypti* population abundance, we have now added Figure 13 and also undertaken additional statistical analyses. The following text was added to the Results and Discussion:

 "There was no clear association between *Wolbachia* establishment in local mosquito populations and seasonal or spatial differences in *Ae. aegypti* abundance in BGS traps. *Ae. aegypti* adult mosquito numbers in BGS traps were significantly (P<0.05) higher during the hot season (May to October) compared with the cool season (November to April) across all sites (Tri Nguyen North *Ae. aegypti *mean catch ratio 1.28 [95% CI 1.18, 1.39]; Center 1.17 [1.07, 1.28]; South 1.42 [1.30, 1.54], Vinh Luong 1.22 [1.22, 1.32]) (Figure 13). In terms of overall mean *Ae. aegypti* numbers in BGS traps during non-release periods, mosquito densities were significantly higher in Tri Nguyen in the South (Mean catch ratio 1.94 [95%CI 1.26, 2.98]) and Center zones (1.69 [1.16, 2.47]) compared with Vinh Luong; however the numbers in Tri Nguyen North were not significantly different to Vinh Luong (1.18 [0.84, 1.68]). Despite the similar and relatively low mosquito densities in Tri Nguyen in the North and in Vinh Luong, and similar seasonal variations in mosquito densities, *Wolbachia* persistence was high across Vinh Luong; whereas its persistence was low in the Tri Nguyen North zone. In contrast, the Tri Nguyen South zone had almost twice the mean numbers of *Ae. aegypti* in BGS traps, compared with Vinh Luong, and *Wolbachia* persistence was high across both sites. Comparisons of *Ae. aegypti* numbers in BGS traps before and after releases indicated that *Ae. aegypti* numbers in Tri Nguyen were higher in the post-release period compared with the pre-release period (Mean catch ratio 1.29 [95% CI 1.18, 1.41]), whereas in Vinh Luong the reverse was observed, with a reduction in numbers during the post-release period (Mean catch ratio 0.87 [95% CI 0.80, 0.96]). Within zone differences in *Ae. aegypti* numbers before and after releases were found in Tri Nguyen, with significantly higher numbers during the post release periods in the Center and South areas ( *Ae. aegypti* mean catch ratios 1.60 [95% CI 1.37, 1.88] and 1.33 [1.15, 1.55], respectively), but not in the North zone (1.04 [0.89, 1.22]). Although these analyses were adjusted for season, they may still be confounded by seasonality as the pre-release monitoring period in both locations was very short (4-5 months). Not surprisingly, the mean numbers of *Ae. aegypti* in BGS traps during the releases were higher than the numbers during non-release periods in both Tri Nguyen (Mean catch ratio 1.82 [95% CI 1.69, 1.96]) and Vinh Luong (1.98 [1.82, 2.15])."

*While temperature effects on WMel are documented from the lab, this study did not conduct any experimental quantification of the role of temperature on WMel infection in the field. Again, it is speculative to assume temperature was the only cause.*

 We acknowledge that there was no experimental data to link field temperatures to reduced Wolbachia prevalence. We have added the text below to the Results and Discussion.

 "Although we did not measure the temperatures in water containers in the field, our study of maternal transmission in field containers set during the two consecutive hot seasons confirm the loss of *w*Mel infection in the progeny of *Wolbachia* infected female mosquitoes. This seasonal loss of *w*Mel infection in *Ae. aegypti* mosquitoes may be due to high temperatures in these larval habitats. Several studies have demonstrated that temperatures >35°C significantly reduce *Wolbachia* infections in *Ae. aegypti* larvae, especially younger instars (Ross et al. 2017, 2019, 2020; Ulrich et al. 2016). Detailed entomological surveys on Tri Nguyen found that large water storage tanks and jars were responsible for over 90% of larval and pupal production (Jeffrey et al. 2009). While temperatures in nearby Nha Trang indicate air temperatures in summer only occasionally exceed 35°C, it is likely that tanks and jars exposed to sunshine would be subject to solar gain and water within them could heat up. Heating of water in containers exposed to even partial sunshine can result in spikes in temperature > 35°C, and concurrent significant loss in *w*Mel density (Ross et al. 2019). Indeed, the amount of water inside tanks and jars may be at a minimum just when temperatures peak in the late dry season. Water in these tanks may heat well beyond 35°C and cure *w*Mel infections. Thus, it is critical that seasonal temperatures and *w*Mel levels in *Ae. aegypti* be monitored in a range of containers in shade and sunlight during the dry season."

*While there is evidence of WMel infection presence, how about WMel density? Why are not loads of WMel in mosquitoes reported as in other WMP studies?*

 We have added Figure 12 showing *Wolbachia* density in *Ae. aegypti* mosquitoes collected in BGS traps from Tri Nguyen and Vinh Luong, and Table 5 showing the correlation between *w*Mel density in weekly collections of *Ae. aegypti* and the current and lagged mean daily temperature measured in households in Tri Nguyen and Vinh Luong.

 The following text was added to the Methods section:

 "The density of *Wolbachia *in *Ae. aegypti *caught over time was summarized as the median relative density value in *Ae. aegypti *caught each week during the post-release period, in each of the three Tri Nguyen areas (north, central, south) and in Vinh Luong. A distributed lag linear regression model was used to fit weekly median *Wolbachia *density to the mean daily temperature measured inside houses. The model included values of weekly mean daily temperature lagged up to 5 weeks for the three Tri Nguyen areas and 4 weeks for Vinh Luong, with maximum lag determined using the Akaike Information Criterion (AIC). Newey-West standard errors were calculated to account for autocorrelation in weekly median *Wolbachia *density, with maximum lag 9 for the three Tri Nguyen areas and maximum lag 5 for Vinh Luong."

 We have also inserted the following text in the Results and Discussion:

 " *Wolbachia* density in *Ae. aegypti* mosquitoes collected in BGS traps varied seasonally across both Tri Nguyen and Vinh Luong (Figure 12). There was a significant negative correlation between the weekly median *Wolbachia* density in mosquitoes trapped during the post-release period and the weekly mean daily temperatures measured in households, in each region of Tri Nguyen and in Vinh Luong (Table 5). This association was significant for both male and female *Wolbachia*-infected mosquitoes, but was much more pronounced in females. Associations were strongest when lagged temperature values of up to 5 weeks were included in the model for Tri Nguyen and up to 4 weeks for Vinh Luong."

 Minor comments:

*The figures show mean numbers of mosquitoes but exclude the variability around the mean. Report standard errors or other measure.*

 Figures 6 and 7 show *Wolbachia* infection prevalence in mosquitoes, not the mean numbers of mosquitoes (total positives / total number tested - blue line, median *Wolbachia* prevalence in mosquitoes per BG trap collection - red line, interquartile range - box plots). In response to Reviewer 1 comment, we have added Figure 13 that shows the Mean (+/- standard deviation [SD]) numbers of *Aedes aegypti* mosquitoes collected in BGS traps per week.

 The authors made an additional, minor correction to the ms. In the *Wolbachia* screening section in the Methods, the following were corrected:

 " either TM513 (primers F: 5’-CAAATTGCTCTTGTCCTGTGG -3’, R: 5’-GGGTGTTAAGCAGAGTTACGG-3’ and probe 5’-LC640 -TGAAATGGAAAAATTGGCGAGGTGTAGG-iowaBlack-3’) or"

 "TM513 primers and probe were used for Tri Nguyen samples to keep consistency with primary results and WSP primers and probe was used for Vinh Luong ward to reduce the possible effect of gene copy number variation in comparison with WS0513 gene."

 The underlying data for Figures 12 and 13 have been uploaded to Figshare:

 Ryan, Peter (2021): Wolbachia density data. figshare. Dataset. https://doi.org/10.6084/m9.figshare.17427479.v1

 Ryan, Peter (2021): TN BGS Ae. aegypti Abundance. figshare. Dataset. https://doi.org/10.6084/m9.figshare.17427434.v1 

 Ryan, Peter (2021): VL BGS Ae. aegypti Abundance. figshare. Dataset. https://doi.org/10.6084/m9.figshare.17427470.v1

# Peer review: Reviewer response for version 1

**DOI:** 10.21956/gatesopenres.14590.r31210

**Published:** 2021-10-15

**Authors:** Zhiyong Xi

**Affiliations:** 1 Department of Microbiology and Molecular Genetics, Michigan State University, East Lansing, Minnesota, USA

Hien *et al.* report different dynamics of *Wolbachia *strain wMel after being released into two field sites in Vietnam, with loss of wMel in two areas of one site and maintenance of high infection in another site. In both sites, a decrease of Wolbachia infection frequency was associated with elevated temperature conditions. However, this high temperature alone can not explain the loss of infection, indicating presence of other factors influencing the local establishment of Wolbachia infection. These discoveries can significantly advance our knowledge of Wolbachia dynamic in field settings and across seasons, support the notion that various Wolbachia strain may be required for different locations when Wolbachia is deployed globally for mosquito-borne disease control. Below are some minor points:

   

 Data show that more mosquitoes were released in Tri Nguyen (32.4 mosquito/house) than in Vinh Luong (11.5 mosquitoes/house) and it took longer time in Tri Nguyen (27 weeks) than in Vinh Luong (17 weeks) to reach a similar infection frequency (~80%). This may indicate that the mosquito population in Tri Nguyen is more difficult for Wolbachia to invade and spread than Vinh Luong. In addition to abiotic factors, difference in mosquito density, population structure and distribution in two release sites may also contribute to the above observation. It will be helpful if the authors can provide some baseline mosquito data before release in these two sites. For example, is it possible that the original mosquito density in Tri Nguyen is much higher than in Vinh Luong such that more release is required in the former? Or whether there is any difference in mosquito populations between North/Central and South Tri Nguyen?

 The authors are suggested to use the same title, mosquitoes released per house per week, for the first Y-axis of both Figures 4 and 5 such that they can be easily compared.

 Figure 6, as no data between June 2019 and April 2021, it is better to put a break in the X-axis to avoid misleading. Same for Figure 7 between Dec 2019 and Nov 2020.

 Figure 6 and 7, it is likely that both temperature and dry condition contribute to loss of Wolbachia infection. The authors are suggested to provide rainfall or humidity data in parallel with temperature in the figure.

 Figure 8 shows that, different from other years, there was a large number of BG traps without *Aedes aegypti* detected in Dec 2016 and July 2017 which occurred mainly in north and center areas and coincides with a large drop of infection frequency below the threshold in these two locations. From Figure 8 and 9, there appears to be a much lower mosquito density in 2019 and 2021 as compared to 2014 and 2015. The authors are suggested to confirm and/or discuss about these observations.  

 There is redundant description in a number of places. Below are two examples.  

1. One in the introduction and another in the discussion (repeat twice): a 69% reduction in dengue incidence, a 56% reduction in chikungunya incidence and a 37% reduction in Zika incidence.
2. One in page 6 and another in page 10 (repeat twice): After 24 h a human blood meal was provided and individual females that appeared fully engorged were placed into individual oviposition cups. … After 24 hours the number of hatched larvae were recorded. Larvae were reared until they reached II-IV instar then transferred to 70% ethanol.

Is the work clearly and accurately presented and does it cite the current literature?

Yes

If applicable, is the statistical analysis and its interpretation appropriate?

Not applicable

Are all the source data underlying the results available to ensure full reproducibility?

Yes

Is the study design appropriate and is the work technically sound?

Yes

Are the conclusions drawn adequately supported by the results?

Yes

Are sufficient details of methods and analysis provided to allow replication by others?

Yes

Reviewer Expertise:

Wolbachia, mosquito, medical entomology, dengue

I confirm that I have read this submission and believe that I have an appropriate level of expertise to confirm that it is of an acceptable scientific standard.

# Response

**Published:** 2021-12-28

**Authors:** Peter Ryan

**Affiliations:** World Mosquito Program, Vietnam

*Data show that more mosquitoes were released in Tri Nguyen (32.4 mosquito/house) than in Vinh Luong (11.5 mosquitoes/house) and it took longer time in Tri Nguyen (27 weeks) than in Vinh Luong (17 weeks) to reach a similar infection frequency (~80%). This may indicate that the mosquito population in Tri Nguyen is more difficult for Wolbachia to invade and spread than Vinh Luong. In addition to abiotic factors, difference in mosquito density, population structure and distribution in two release sites may also contribute to the above observation. It will be helpful if the authors can provide some baseline mosquito data before release in these two sites. For example, is it possible that the original mosquito density in Tri Nguyen is much higher than in Vinh Luong such that more release is required in the former? Or whether there is any difference in mosquito populations between North/Central and South Tri Nguyen?*

*The authors are suggested to use the same title, mosquitoes released per house per week, for the first Y-axis of both Figures 4 and 5 such that they can be easily compared.*

 This has been corrected. Figure 5 has been revised with the Y-axis now showing "Mean numbers of *Wolbachia* mosquitoes released per house per week", which is consistent with Figure 4.

*Figure 6, as no data between June 2019 and April 2021, it is better to put a break in the X-axis to avoid misleading. Same for Figure 7 between Dec 2019 and Nov 2020.*

 These have been corrected. Figures 6 and 7 have been revised and now include breaks in the *Wolbachia *frequency curves (Mean and Median) to indicate there was no monitoring data during these periods. The X-Axes have been retained as there were, as the graphs contain temperature and rainfall data throughout the entire study period. Text has also been added to the figure captions to indicate that there was no *Wolbachia* monitoring data during the respective periods for Tri Nguyen (Figure 6) and Vinh Luong (Figure 7).

*Figure 6 and 7, it is likely that both temperature and dry condition contribute to loss of Wolbachia infection. The authors are suggested to provide rainfall or humidity data in parallel with temperature in the figure.*

 Figures 6 and 7 have been revised and now include weekly rainfall data.

*Figure 8 shows that, different from other years, there was a large number of BG traps without Aedes aegypti detected in Dec 2016 and July 2017 which occurred mainly in north and center areas and coincides with a large drop of infection frequency below the threshold in these two locations. From Figure 8 and 9, there appears to be a much lower mosquito density in 2019 and 2021 as compared to 2014 and 2015. The authors are suggested to confirm and/or discuss about these observations.*

 With thank the reviewer for this comment. We have inserted an additional figure (Figure 13) that shows the Mean +/- standard deviation numbers of *Ae. aegypti* per BGS trap per week (Note: *Wolbachia* infection screening was not undertaken on all sampling weeks, so there is additional weekly mosquito density data in Figure 13, compared to the weeks with *Wolbachia* screening data in Figures 6-10.

 Additional statistical analyses were undertaken to determine whether there were differences in the mean *Ae. aegypti* numbers in BGS traps between pre-release and post-release periods in Tri Nguyen and Vinh Luong. The following has been added to the methods section:

 "Statistical analysis

 The number of *Ae. aegypti* mosquitoes caught over time was summarized as the mean count per BGS trap per week, in each of the three Tri Nguyen areas (north, central, south) and in Vinh Luong. Mixed-effects negative binomial regression was used to compare the number of *Ae. aegypti* caught per BGS trap per week, between pre- and post-release periods, with the inclusion of BGS trap as a random effect to account for clustering at the trap level. All analyses included a binary indicator for hot/cool season as a covariate (hot season = May - October; cool season November - April). The count ratio produced by the negative binomial regression model is the ratio of the mean number of *Ae. aegypti* caught per trap per week between areas or between release vs non-release periods. Data was analyzed by release status: pre-release, during release, and post-release, and between sites for the total observation period. Analysis of the total observation period was done with the inclusion of an indicator for release status as a covariate."

 The following was added to the discussion section (along with additional analyses in response to reviewer 2 comments):

 "There was no clear association between *Wolbachia* establishment in local mosquito populations and seasonal or spatial differences in *Ae. aegypti* abundance in BGS traps. *Ae. aegypti* adult mosquito numbers in BGS traps were significantly (P<0.05) higher during the hot season (May to October) compared with the cool season (November to April) across all sites (Tri Nguyen North *Ae. aegypti *mean catch ratio 1.28 [95% CI 1.18, 1.39]; Center 1.17 [1.07, 1.28]; South 1.42 [1.30, 1.54], Vinh Luong 1.22 [1.22, 1.32]) (Figure 13). In terms of overall mean *Ae. aegypti* numbers in BGS traps during non-release periods, mosquito densities were significantly higher in Tri Nguyen in the South (Mean catch ratio 1.94 [95%CI 1.26, 2.98]) and Center zones (1.69 [1.16, 2.47]) compared with Vinh Luong; however the numbers in Tri Nguyen North were not significantly different to Vinh Luong (1.18 [0.84, 1.68]). Despite the similar and relatively low mosquito densities in Tri Nguyen in the North and in Vinh Luong, and similar seasonal variations in mosquito densities, *Wolbachia* persistence was high across Vinh Luong; whereas its persistence was low in the Tri Nguyen North zone. In contrast, the Tri Nguyen South zone had almost twice the mean numbers of *Ae. aegypti* in BGS traps, compared with Vinh Luong, and *Wolbachia* persistence was high across both sites. Comparisons of *Ae. aegypti* numbers in BGS traps before and after releases indicated that *Ae. aegypti* numbers in Tri Nguyen were higher in the post-release period compared with the pre-release period (Mean catch ratio 1.29 [95% CI 1.18, 1.41]), whereas in Vinh Luong the reverse was observed, with a reduction in numbers during the post-release period (Mean catch ratio 0.87 [95% CI 0.80, 0.96]). Within zone differences in *Ae. aegypti* numbers before and after releases were found in Tri Nguyen, with significantly higher numbers during the post release periods in the Center and South areas ( *Ae. aegypti* mean catch ratios 1.60 [95% CI 1.37, 1.88] and 1.33 [1.15, 1.55], respectively), but not in the North zone (1.04 [0.89, 1.22]). Although these analyses were adjusted for season, they may still be confounded by seasonality as the pre-release monitoring period in both locations was very short (4-5 months). Not surprisingly, the mean numbers of *Ae. aegypti* in BGS traps during the releases were higher than the numbers during non-release periods in both Tri Nguyen (Mean catch ratio 1.82 [95% CI 1.69, 1.96]) and Vinh Luong (1.98 [1.82, 2.15])."

*There is redundant description in a number of places. Below are two examples.*

*One in the introduction and another in the discussion (repeat twice): a 69% reduction in dengue incidence, a 56% reduction in chikungunya incidence and a 37% reduction in Zika incidence.*

 This has been corrected. The following has been deleted from the discussion "Aggregate across the whole intervention area, the *w*Mel deployments were associated with a 69% reduction in dengue incidence, a 56% reduction in chikungunya incidence and a 37% reduction in Zika incidence."

*One in page 6 and another in page 10 (repeat twice): After 24 h a human blood meal was provided and individual females that appeared fully engorged were placed into individual oviposition cups. … After 24 hours the number of hatched larvae were recorded. Larvae were reared until they reached II-IV instar then transferred to 70% ethanol.*

 This has been corrected. The following has been deleted from the methods section "After 24 h a human blood meal was provided and individual females that appeared fully engorged were removed from the cage and placed into individual oviposition cups. Each cup was lined with a piece of filter paper as a medium for oviposition. After 24 hrs following oviposition, the female mosquitoes were collected and stored in 70% ethanol, and the eggs were counted and then conditioned for three days, prior to hatching in water containing a small amount of fish food (JBL NovoTab, Neuhofen, Germany, Product number 302300). After 24 hours the number of hatched larvae were recorded. Larvae were reared until they reached II-IV instar then transferred to 70% ethanol. Adult females and progeny (n=10-20) were processed for Wolbachia infection using a Taqman qPCR assay (Dar et al., 2008; O’Neill et al., 2019; Yeap et al., 2014)."

 The above text was replaced with the following "Mosquitoes were blood fed and the progeny were processed as described previously, except immatures were fed on JBL NovoTab fish food (JBL NovoTab, Neuhofen, Germany, Product number 302300)."
